# Cost-effectiveness of population-based screening for chronic obstructive pulmonary disease in China: a simulation modeling study

**DOI:** 10.1016/j.lanwpc.2024.101065

**Published:** 2024-04-29

**Authors:** Qiushi Chen, Yiwen Fan, Ke Huang, Wei Li, Pascal Geldsetzer, Till Bärnighausen, Ting Yang, Chen Wang, Simiao Chen

**Affiliations:** a The Harold and Inge Marcus Department of Industrial and Manufacturing Engineering, The Pennsylvania State University, University Park, PA, USA; b Heidelberg Institute of Global Health, Faculty of Medicine and University Hospital, Heidelberg University, Heidelberg, Germany; c Department of Pulmonary and Critical Care Medicine, Center of Respiratory Medicine, China-Japan Friendship Hospital, Beijing, China; d Division of Primary Care and Population Health, Department of Medicine, Stanford University School of Medicine, Stanford, CA, USA; e Chinese Academy of Medical Sciences and Peking Union Medical College, Beijing, China; f National Clinical Research Center for Respiratory Diseases, Beijing, China; g Chinese Academy of Engineering, Beijing, China

**Keywords:** Chronic obstructive pulmonary disease, Population-based screening, Microsimulation, Cost-effectiveness analysis, Public health policy

## Abstract

**Background:**

China has the highest disease burden of chronic obstructive pulmonary disease (COPD) in the world; however, the diagnosis rate remains low. Screening for COPD in the population may improve early diagnosis and long-term health outcomes for patients with COPD. In this study, we aimed to evaluate the cost-effectiveness of population-based COPD screening policies in China.

**Methods:**

We developed a microsimulation model that simulated incidence, natural history, and clinical management of COPD over a lifetime horizon among the general population aged 35–80 years in China. We evaluated population-based screening policies with different screening methods (one-step with COPD Screening Questionnaire or two-step with additional portable spirometer test) and frequencies (one-time or every 1–10 years). We calculated the incremental cost-effectiveness ratio (ICER) of the screening policies compared with the status quo (without screening) and identified the most cost-effective screening policy. Scenario and sensitivity analyses were performed to assess the impact of key parameters and the robustness of model results.

**Findings:**

Compared with the status quo, all population-based COPD screening policies were cost-effective with estimated ICERs ranging between $8034 and $13,209 per quality-adjusted-life-year (QALY), all under the willingness-to-pay value of $38,441/QALY (three times China's gross domestic product per capita). A total of 0.39%–8.10% of COPD-related deaths and 0.58%–2.70% of COPD exacerbations were projected to be averted by COPD screening. Among all screening policies, annual two-step screening was the most cost-effective. Improving the linkage from screening to diagnosis and treatment could further increase population health benefits and the cost-effectiveness of COPD screening.

**Interpretation:**

Population-based screening for COPD could be cost-effective in China. Offering public programs for COPD screening similar to existing preventive health services for other chronic diseases could be a promising strategy to improve population health outcomes and mitigate the disease burden of COPD in China.

**Funding:**

10.13039/100005156Alexander von Humboldt Foundation, 10.13039/501100001809National Natural Science Foundation of China, 10.13039/501100005150CAMS Innovation Fund for Medical Science, 10.13039/501100019069Chinese Academy of Engineering project, and Horizon Europe.

**Research in context**

**Evidence before this study**

To facilitate early diagnosis of chronic obstructive pulmonary disease (COPD), international guidelines have recommended case-finding strategies among the targeted high-risk population in primary care settings, whereas screening in the asymptomatic population has not been recommended due to a lack of evidence of clinical benefits. China has the highest disease burden of COPD in the world with a very low diagnosis rate, representing a disease landscape that is markedly different from that in the countries in which the evidence for established clinical guidelines was mainly generated. Given the lack of China-specific evidence in this domain, the value of a population-based COPD screening strategy in China is worthy of close examination. We searched PubMed, Embase, and Google Scholar between January 1, 2000, and May 27, 2023, with no language restrictions, for the search terms “COPD” (or “chronic obstructive pulmonary disease”) and “screening” (or “diagnosis”) and “China” (or “Chinese”) and “cost-effectiveness” (or “economic evaluation” or “cost-utility”) to identify published economic evaluations of population-based COPD screening policies in China. We also explored economic evaluation studies of COPD screening in other countries using the same terms but without specifying “China” (or “Chinese”). We found several studies that evaluated COPD screening for specific high-risk populations (e.g., age >45 years, smokers, or patients with chronic bronchitis) or focused on case-finding strategies in primary care; however, no previous study had comprehensively assessed the cost-effectiveness of population-based screening in the setting of China. Additionally, no studies were found that assessed the cost-effectiveness of population-based COPD screening in other country settings.

**Added value of this study**

To our knowledge, this is the first study evaluating the cost-effectiveness of population-based screening for COPD in China. While screening for COPD is not recommended for the asymptomatic population in several clinical guidelines primarily focused on developed countries, we found that population-based screening for COPD could be cost-effective for the general population aged 35 years and above in China compared with the status quo without a systemic screening program. The cost-effectiveness of COPD screening was robust across screening frequencies (ranging from one-time screening to annual screening) and screening methods (including one-step screening based on COPD Screening Questionnaire [COPD-SQ] and two-step screening with COPD-SQ followed by a portable spirometer test). One-step screening policies yielded more quality-adjusted life years and had higher costs than two-step screening policies, but these differences were not substantial. Among all screening policies, two-step annual screening was deemed the most cost-effective. We also found that COPD screening becomes more cost-effective with improved linkage to follow-up diagnosis and treatment following positive screenings.

**Implications of all the available evidence**

China has the highest burden of COPD in the world, yet awareness of the disease remains low. Effective prevention and intervention strategies are urgently needed to reduce COPD's health and economic burdens at the population level. Population-based screening for COPD is a cost-effective strategy in China. Compared with one-step screenings, two-step screenings are more selective in referring patients to follow-up diagnosis, which could be more practical in resource-limited settings such as rural towns in China, as it would require fewer patients to travel to large hospitals in urban areas for follow-up diagnosis. Improving linkage to follow-up confirmatory diagnosis and timely treatment can enhance the cost-effectiveness of screening. Given the robust cost-effectiveness findings, policymakers should consider implementing public health programs to facilitate population-based COPD screening, such as covering COPD screening services in national public health programs. Such policies may improve COPD patients' awareness of the disease and access to care, thereby improving population health outcomes.

## Introduction

Chronic obstructive pulmonary disease (COPD) is a common chronic illness that is characterized by persistent respiratory symptoms and airflow limitation.^1^^,^^2^ Patients with COPD may experience dyspnea, cough, or sputum production; some may experience exacerbations, resulting in a sharp reduction in lung function and quality of life.^3^ COPD is the third-leading cause of death globally.^4^ Thought to be due to a high prevalence of cigarette smoking and high levels of air pollution, China's COPD prevalence is higher than in most other countries,^5^, ^6^, ^7^ estimated at 8.6% in adults aged 20 years and above and 13.7% in adults aged 40 years and above according to the China Pulmonary Health (CPH) study.^8^ Each year, over one million people die from COPD in China, more than in any other country in the world.^9^^,^^10^ In addition to exacting a major health burden, COPD also carries a significant economic burden and is projected to cost China $1.4 trillion from 2020 to 2050, accounting for about one third of the global economic burden of COPD.^11^

Notwithstanding its major health and economic burdens, COPD can be prevented and treated. Early and accurate diagnosis is thought to be important for effective management of COPD.^12^^,^^13^ Previous studies have demonstrated that early diagnosis of COPD followed by timely treatment can improve health outcomes by slowing the decline of lung function^14^ and reducing the risk of exacerbations.^15^ However, COPD remains severely underdiagnosed among the population in China, with less than 3% of COPD patients estimated to be aware of their condition.^8^ One study found that among patients who were aware of their COPD diagnosis, most had already progressed to a moderate or severe stage of the disease by the time they sought diagnosis following symptom onset,^16^ and thus had missed the opportunity for early intervention to improve their long-term health outcomes. On the other hand, a spirometry-based and nationally representative cross-sectional study in China found that over 90% of all patients with COPD were still in the mild-to-moderate stage of the disease.^17^ Early identification of these patients, potentially through a systematic screening policy, is crucial for facilitating timely treatment and relieving COPD's burden on society.

Existing clinical guidelines offer little in the way of direction on how COPD screening should be conducted in China. The US Preventive Services Task Force (USPSTF)^18^ and the UK National Screening Committee^19^ do not recommend screening for COPD among asymptomatic patients due to a lack of definitive evidence of improved clinical health outcomes from screening, and several other international guidelines^1^^,^^20^ emphasize the importance of case-finding in primary care settings rather than population-based screening. China has recently launched a pilot nationwide population-based screening program, the Enjoying Breathing Program,^21^ which uses a combination of a COPD Screening Questionnaire (COPD-SQ, a questionnaire validated for the Chinese population^22^) and portable spirometry testing to facilitate early identification and intervention for individuals with COPD. While there have been several economic evaluation studies of targeted COPD screening in China that primarily focused on high-risk populations,^23^^,^^24^ the cost-effectiveness of population-based COPD screening policies remains unknown. Such data are much needed for supporting effective utilization of population health resources to mitigate the high burden of COPD in the country.

To fill this gap, we aimed to evaluate the cost-effectiveness of COPD screening policies for the general population in China using a simulation modeling approach. While this modeling approach has been extensively utilized to inform screening policies for cancer,^25^, ^26^, ^27^ infectious diseases,^28^^,^^29^ and other chronic diseases,^30^, ^31^, ^32^ its application to COPD screening has been limited. Our objective was to project health and economic outcomes for the overall population under population-based COPD screening policies with different combinations of screening methods and frequencies and to identify the most cost-effective policy. This study seeks to inform the development of real-world COPD screening programs.

## Methods

**Fig. 1:**
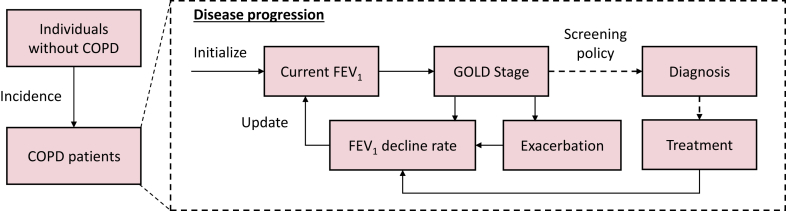
**Schematic of the COPD microsimulation (COPD-SIM) model.** The model simulated individual-level changes in new incidence of COPD, decline in lung function (measured by forced expiratory volume in 1 second, *FEV*_*1*_), awareness of disease diagnosis, treatment status, and clinical exacerbation. The annual decline rate in lung function was jointly determined by the current clinical stage of COPD (measured in GOLD Stage), treatment, and exacerbations.

We developed the COPD microsimulation (COPD-SIM) model, a detailed individual-level simulation model for COPD, to represent the natural history and clinical management of the disease. The model simulates disease progression as changes in lung function, disease exacerbation, and clinical care (e.g., diagnosis, treatment) for each patient with a model cycle of three months over a lifetime horizon (Fig. 1). We projected the total discounted quality-adjusted life years (QALYs) and cost for a general population aged 35–80 years in China under different COPD screening policies and identified the most cost-effective policies. We followed the Consolidated Health Economic Evaluation Reporting Standards (CHEERS) guidelines for reporting our study design and outcomes.^33^ In the following, we describe the key components of COPD-SIM, summarize key model parameters in Table 1, and provide more details on model implementation and parameter estimation in Supplements S1 and S2.

**Table 1:** Key input parameters for the COPD-SIM model.

Parameter	Base case value	Source
COPD epidemiology parameters		
Prevalence rate of COPD, % (male, female)		CPH study,^8^Supplement S1.1
35–39 years^a^	(1.86, 1.79)	
40–49 years	(7.45, 3.14)	
50–59 years	(15.57, 5.96)	
60–69 years	(27.08, 11.58)	
70–80 years	(42.96, 20.27)	
Incidence rate of COPD, per 100,000 population (male, female)		GBD study,^10^ CPH study,^8^Supplement S1.1
35–39 years	(50.42, 48.50)	
40–49 years	(165.64, 69.84)	
50–59 years	(442.46, 169.44)	
60–69 years	(1053.21, 450.33)	
70–80 years^b^	(5056.24, 2385.55)	
*FEV*_*1*_% predicted for the initial population (normally distributed)	Male: μ = 0.797, σ = 0.206 Female: μ = 0.835, σ = 0.206	^34^Supplement S1.2
*FEV*_*1*_% predicted for the new incidence (normally distributed)	Male: μ = 1.014, σ = 0.906 Female: μ = 1.034, σ = 0.107	^34^
Diagnosis rate in the initial COPD cohort, %		CPH study^34^
GOLD Stage 1: mild	0.63%	
GOLD Stage 2: moderate	2.00%	
GOLD Stage 3: severe	12.87%	
GOLD Stage 4: very severe	21.04%	
Annual decline of *FEV*_*1*_, ml/year (normally distributed)		Supplement S1.2
GOLD Stage 1: mild	μ = 40, σ = 5	SAPALDIA study^35^
GOLD Stage 2: moderate	μ = 60, σ = 5	TORCH study^15^
GOLD Stage 3: severe	μ = 56, σ = 5	
GOLD Stage 4: very severe	μ = 34, σ = 5	
Annual rate of exacerbation		^36^
Non-severe		
GOLD Stage 1: mild	0.71	
GOLD Stage 2: moderate	1.01	
GOLD Stage 3: severe	1.39	
GOLD Stage 4: very severe	1.82	
Severe		
GOLD Stage 1: mild	0.11	
GOLD Stage 2: moderate	0.16	
GOLD Stage 3: severe	0.22	
GOLD Stage 4: very severe	0.28	
Hazard ratio of mortality for COPD patients		^37^Supplement S1.2
GOLD Stage 1: mild	1.2	
GOLD Stage 2: moderate	1.6	
GOLD Stage 3: severe	2.7	
GOLD Stage 4: very severe	2.7	
COPD treatment effectiveness^c^		Supplement S1.4
Reduction in *FEV*_*1*_ annual decline rate (ml)	73–201	
Relative risk in exacerbation	0.62–0.92	
Relative risk in mortality	0.71–0.99	
Screening		
COPD screening questionnaire (COPD-SQ)		^38^
Sensitivity	0.57	
Specificity	0.82	
Portable spirometer		^39^
Sensitivity	0.85	
Specificity	0.85	
Health-related quality of life		^40^Supplement S1.5
GOLD Stage 1: mild	0.806	
GOLD Stage 2: moderate	0.767	
GOLD Stage 3: severe	0.704	
GOLD Stage 4: very severe	0.616	
Cost (US$)		
Screening and diagnostic cost		Supplement S1.6
Screening questionnaire administration	0.72	
Portable spirometer test	2.90	
Screening program setup cost (per person)	4.00	
Diagnostic spirometry test	26.93	
Monthly maintenance cost (per person)		^41^
GOLD Stage 1: mild	7.66	
GOLD Stage 2: moderate	24.25	
GOLD Stage 3: severe	34.56	
GOLD Stage 4: very severe	54.04	
Exacerbation cost (per event)		^41^
Non-severe	68.24	
Severe	2987.06	
Monthly treatment cost (per person)	20.21–104.58^c^	^23^

*Abbreviations*: CPH, China Pulmonary Health; GBD, Global Burden of Disease; SAPALDIA, Swiss Study on Air Pollution and Lung Diseases in Adults; FEV_1_, forced expiratory volume in 1 s.

a We used the prevalence estimates for the 20- to 39-year age group originally reported in the CPH study.^8^

b We used the incidence rate estimate for the 70- to 85-year age group originally reported in the GBD study.^10^

c Since the parameters depended on treatment type, only ranges were presented in this table. See Supplement S1.4 and full parameter table in Supplement S2 for more details of treatment-specific parameters.

### Simulation cohort

The simulation model initialized a cohort of one million individuals aged between 35 and 80 years following the sex and age distributions of the general population in China.^42^ The lower age limit of 35 years was selected to be comparable to the starting age for other screening programs (e.g., for hypertension) covered by the Basic Public Health Service program in China.^43^ The COPD patient cohort was initialized based on age- and sex-specific prevalence in the general population from the nationally representative cross-sectional CPH study^8^ (Supplement S1.1). Individuals without COPD from the general population could develop the condition in each model period at the risk calculated from age- and sex-specific incidence rates.^10^

### Natural history model for disease progression

We simulated the disease progression process by tracking the decline of a COPD patient's lung function characterized by the forced expiratory volume in 1 second (*FEV*_*1*_) and the value of *FEV*_*1*_*% predicted* (representing the degree of airway obstruction) over time (Fig. 1). The value of *FEV*_*1*_*% predicted* determined the severity stage according to the GOLD criteria (i.e., percentage of predicted *FEV*_*1*_ ≥80%, 50–79%, 30–49%, and <30% corresponding to GOLD stages 1–4, respectively).^1^ Changes in the GOLD stage would in turn affect the annual decline rate of the *FEV*_*1*_ value. In each period, all individuals in the simulation model were at age-and-sex-specific background mortality estimated from the life table for China,^44^ and COPD patients were at a greater risk of mortality based on their GOLD stage.^45^ More details on the calculation of *FEV*_*1*_ and *FEV*_*1*_*% predicted* values and the estimation of mortality risks are provided in Supplement S1.2.

Exacerbation is an important clinical event in the disease course of COPD, resulting in decreased quality of life and additional healthcare costs.^3^^,^^46^ Our model considered two types of exacerbation: *non-severe* exacerbation without hospitalization and *severe* exacerbation requiring hospital admission, with different frequencies by GOLD stage. The *FEV*_*1*_ annual decline rate was estimated to increase by 95.7% after an exacerbation (either non-severe or severe) based on the results of the UPLIFT study^47^; these increases were assumed to last for 12 months considering that data for longer time horizons from the clinical study were limited (Supplement S1.2).

### Diagnosis and treatment

In the absence of systemic screening efforts (i.e., the status quo), COPD patients are typically diagnosed when seeking care due to symptoms. We modeled symptom-based diagnoses to occur with relatively low probability in each period, which we calibrated based on the observed COPD awareness rates by GOLD stage from the CPH study^8^ (Supplement S1.3). To capture undertreatment following diagnosis, we used a conservative treatment rate of 30%, as observed in the CPH study,^34^ among diagnosed patients regardless of their prior history of screening. We considered five common types of long-acting treatment for COPD, with the treatment distributions depending on the patients' GOLD stages (Supplement S1.4). The effects of treatment on reducing the *FEV*_*1*_ decline rate, the exacerbation rate, and the mortality rate were synthesized from multiple clinical studies (see estimation details in Supplement S1.4). Since most clinical studies had limited observation time and data for long-term treatment effects were lacking, we assumed that the treatment effect on reducing *FEV*_*1*_ decline rate lasted for 12 months in our simulation model. Treatment was readjusted when a patient's GOLD stage changed.

### Screening policies

**Fig. 2:**
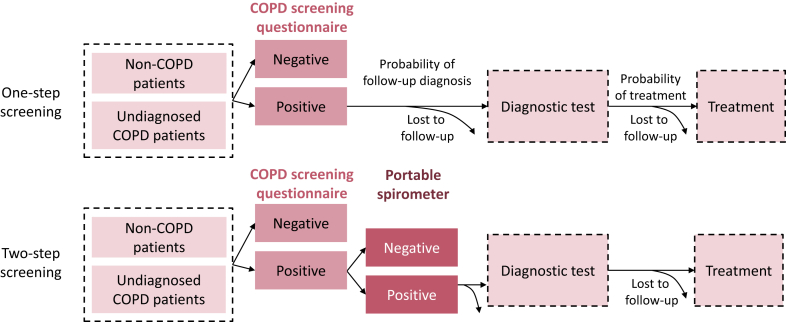
**The processes of the one-step and the two-step screening methods**.

In comparison with the status quo of current clinical practice without COPD screening, we defined a set of population-based COPD screening policies with different configurations of screening methods and frequencies. In particular, we considered two screening methods (Fig. 2): (1) the one-step questionnaire-based screening method in which COPD-SQ was administered to the general population (except for patients already diagnosed with COPD) to identify high-risk individuals for confirmatory testing, and (2) the two-step screening method in which portable spirometry testing was administered for individuals identified as positive by COPD-SQ. The screening frequencies were (1) one-time screening at the beginning of the simulation, (2) every year, (3) every 2 years, (4) every 5 years, and (5) every 10 years.

COPD-SQ was selected as the instrument for the initial screening step for two reasons: First, it was originally developed and validated with adequate accuracy for screening in Chinese population,^38^^,^^48^ second, it had previously been used in the Enjoying Breathing Program,^21^ a pilot, nationwide population-based screening program in China. In the two-step screening method, we added secondary portable spirometer testing within the same visit as questionnaire administration as a practical strategy given that spirometer testing had also previously been implemented for screening in the Enjoying Breathing Program.^21^ The sensitivity and specificity of COPD-SQ and the portable spirometer were obtained from published studies.^38^^,^^39^ To capture inadequate follow-up for confirmatory diagnosis among patients with positive screenings, we assumed a 40% follow-up diagnosis probability based on observed follow-up rates in China.^21^^,^^49^

### Model outcomes

To estimate health-related quality of life, we used the baseline utility by age group for the general population in China,^50^ adjusted it by GOLD stage based on the utility of COPD patients from a meta-analysis,^40^ and accounted for utility reductions due to exacerbation.^3^ For cost outcomes, we took the payer's perspective and considered all direct costs, including the cost of screening, diagnosis, treatment, routine disease maintenance, exacerbation, and other common complications such as pneumonia.^51^ Cost parameters were estimated from public sources including the Beijing Municipal Medical Insurance Bureau open database^52^^,^^53^ and published literature (see estimation details in Supplement S1.6). All costs in the model have been converted to 2022 US dollars.

The primary outcomes of the simulation model included total quality-adjusted life years (QALY) and costs under each screening scenario. Our model also projected the number of COPD-related deaths and exacerbations as secondary outcomes. QALY and cost outcomes were discounted at a 3% annual rate following WHO guidelines for cost-effectiveness analysis.^54^ We calculated the incremental cost-effectiveness ratio (ICER) of each screening policy compared with the status quo and identified the cost-effectiveness frontier consisting of all non-dominated policies. We determined cost-effectiveness using a willingness-to-pay (WTP) value of approximately $38,000 based on three times China's Gross Domestic Product (GDP) per capita in 2022.^55^ We validated our model by verifying that simulation projected outcomes under the status quo were comparable with those from other published modeling studies or clinical studies (Supplement S1.7).

### Scenarios and sensitivity analysis

To understand how linkage to care for COPD diagnosis and treatment could impact the value of COPD screening, we re-evaluated the cost-effectiveness of all screening policies assuming higher probabilities of taking a follow-up diagnostic test after a positive screening outcome and more optimistic post-diagnosis treatment uptake rates that were closer to the values reported in other healthcare settings.^56^, ^57^, ^58^ In addition, we performed one-way sensitivity analyses to assess the impact of individual input parameters on cost-effectiveness outcomes (Supplement S1.8.1). We also performed scenario analyses for the key model parameters of screening accuracy, treatment distribution, and cost (Supplement S1.8.2). To consider the uncertainty of all model input parameters simultaneously, we conducted probabilistic sensitivity analyses (PSA) with 1000 Monte Carlo replications and evaluated the probability of being most cost-effective for each screening policy (Supplement S1.8.3). The microsimulation model was implemented in Python 3.6, and all statistical analyses were performed in R (version 4.0.3).

### Role of the funding source

The funder of the study had no role in the study design, data collection, data analysis, data interpretation, or writing of the report.

## Results

### Base case results

Under the status quo without population-based screening for COPD, our model projected a total of 16,191,816 QALYs over the lifetime of the cohort of one million individuals from the general population of China (Table 2). With population-based COPD screening, the total QALYs increased to 16,194,894–16,210,042 QALYs as screening frequency increased from one-time screening to annual screening. The improvements from one-step to two-step screening policies were marginal. In addition, COPD screening policies with varying methods and frequencies were projected to avert 14,001–64,952 exacerbations (0.58%–2.70% reduction) and 223–4811 deaths (0.39%–8.10% reduction) compared with the status quo.

**Table 2:** Cost-effectiveness of population-based screening policies for chronic obstructive pulmonary disease (COPD) in a cohort of one million individuals over a lifetime horizon.

Screening policy		Health outcomes						Cost outcomes	Cost-effectiveness			
Frequency	Method	Total QALYs for the entire population (millions)	Total QALYs for COPD patients (millions)	Exacerbations, total number	Exacerbations averted (%)	COPD-related deaths, total number	COPD-related deaths averted (%)	Total cost (million US$)	Cost per exacerbation averted (US$)	Cost per death averted (US$)	ICER, screening policy vs. status quo (US$/QALY gained)	ICER, non-dominated policies (US$/QALY gained)
No screening	Status quo	16.192	1.201	2,407,275	–	59,412	–	1374	–	–	–	–
One-time	Two-step	16.195	1.204	2,393,273	0.58	59,178	0.39	1411	2666	159,995	12,128	Dominated
One-time	One-step	16.195	1.205	2,389,691	0.73	59,024	0.65	1418	2541	115,194	13,209	Dominated
Every 10 years	Two-step	16.199	1.208	2,387,576	0.82	57,895	2.55	1430	2866	37,215	8034	8034
Every 10 years	One-step	16.200	1.209	2,383,539	0.99	57,786	2.74	1439	2737	39,968	8212	Dominated
Every 5 years	Two-step	16.201	1.210	2,377,237	1.25	57,517	3.19	1450	2550	40,430	8551	Dominated
Every 5 years	One-step	16.202	1.211	2,372,065	1.46	57,295	3.56	1465	2585	42,997	9217	Dominated
Every 2 years	Two-step	16.206	1.216	2,359,074	2.00	56,632	4.68	1494	2499	43,338	8288	8526
Every 2 years	One-step	16.207	1.217	2,356,091	2.13	55,821	6.04	1512	2699	38,466	8875	Dominated
Every year	Two-step	16.210	1.219	2,345,119	2.58	55,059	7.33	1538	2649	37,831	9267	13,671
Every year	One-step	16.210	1.219	2,342,323	2.70	54,601	8.10	1561	2887	38,981	10,289	49,889

The total cost was projected to be $1374 million under the status quo (Table 2). The two-step screening policy increased the total cost to $1411 million with one-time screening and $1538 million with annual screening. The total cost of one-step screening policies was marginally higher, ranging between $1418 and $1561 million as the screening frequency varied. The higher total cost of one-step screening resulted from the fact that it enabled more patients (including false positives from the screening) to proceed to subsequent diagnosis and treatment. Costs related to managing clinical complications (exacerbation and pneumonia) accounted for the majority (54–64%) of the total cost (Supplement S3, Fig. S5, Table S10), whereas the cost of screening, despite population-wide coverage, accounted for less than 2% of the total cost across all policies.

**Fig. 3:**
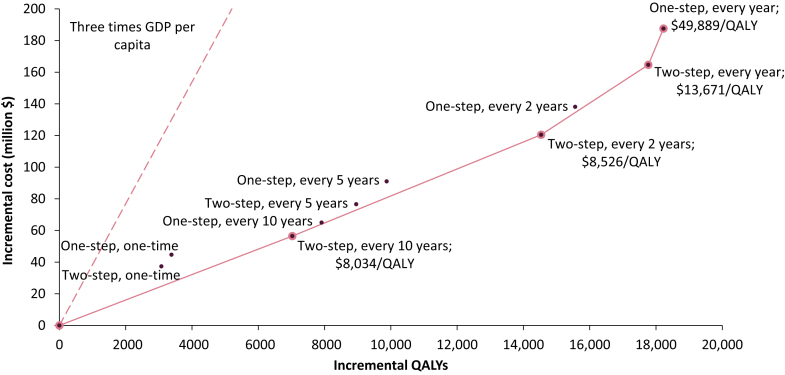
**Cost-effectiveness of all screening policies and the cost-effectiveness frontier**.

Compared with the status quo, all screening policies were deemed cost-effective with estimated ICERs between $8034/QALY and $13,209/QALY, below the WTP value of $38,441/QALY (i.e., three times China's GDP per capita) (Table 2). We identified four non-dominated screening policies on the cost-effectiveness frontier (Fig. 3), including two-step screening every 10 years, two-step screening every 2 years, two-step screening every year, and one-step screening every year. Two-step screening every year was deemed the most cost-effective screening policy with an ICER of $13,671/QALY compared with its precedent non-dominated policy. With a lower WTP value based on one time GDP per capita ($12,813/QALY), the most cost-effective screening policy was two-step screening every 2 years.

### Scenarios of improved linkage to care

**Fig. 4:**
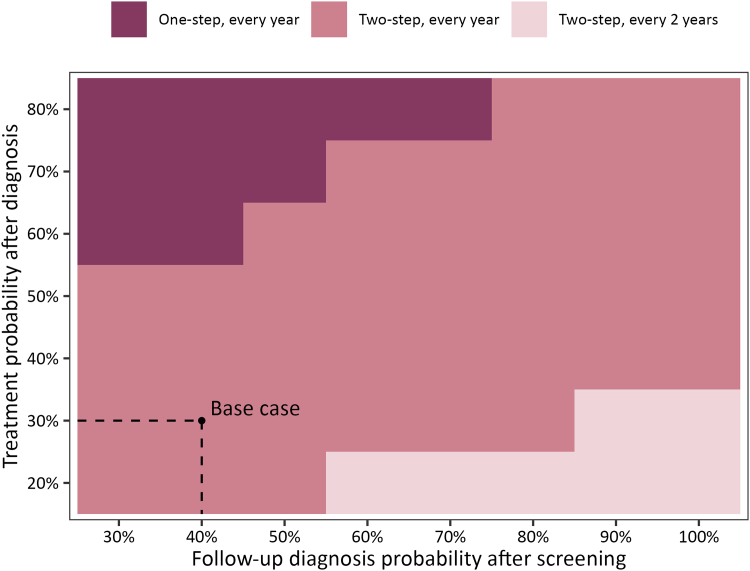
**The most cost-effective screening policies under different linkage to care scenarios with varying follow-up diagnosis and treatment uptake probabilities**.

To understand how the effectiveness and cost-effectiveness of screening policies would change if the linkage to diagnosis and treatment following screening were improved from the level in the base case setting, we re-evaluated the screening policies by varying the probabilities of follow-up diagnosis and treatment uptake (Fig. 4). When only the follow-up diagnosis probability was increased from 30% to 100%, the most cost-effective policy became less frequent, changing from two-step screening every year to two-step screening every two years; when the treatment probability was increased to above 50%, the most cost-effective policy changed from two-step to one-step screening every year. Overall, the screening policies became more cost-effective with lower ICERs when linkage to care was improved (Supplement S3, Figs. S6 and S7, Table S11). Although the most cost-effective policy remained unchanged (two-step screening every year) when both follow-up diagnosis and treatment probabilities increased from their base case values to 100% and 80%, respectively, its ICER decreased from $12,458/QALY to $7800/QALY, and its incremental QALYs compared with the status quo increased nearly eight-fold from 8100 to 68,807 QALYs.

### Sensitivity analysis

The one-way sensitivity analysis showed that variations in all parameters did not substantially impact the cost-effectiveness of two-step screening every year, the most cost-effective screening policy from the base case (Supplement S3, Figs. S8 and S9, Table S12). Parameters with a high impact on the ICERs included the effect of treatment on the exacerbation rate, the mortality hazard ratios for COPD patients, the assumption of resetting the effect of treatment on lung function decline when switching treatment, the effect of treatment on mortality, the disutility of severe exacerbation, and the utility multiplier for each COPD stage. Screening accuracy, treatment distribution, and cost had limited impact on the base case cost-effectiveness findings (Supplement S3, Tables S13–15). The cost-effectiveness acceptability curves from the PSA showed that two-step screening every year had the highest probability of being most cost-effective when WTP ranged between $12,900 and $50,600 (approximately one to four times GDP per capita) (Supplement S3, Fig. S10). If WTP increased above this range, one-step screening every year had a dominating probability of being the most cost-effective screening policy.

## Discussion

COPD poses a major public health challenge in China. Although the disease can be effectively managed with early diagnosis and intervention, awareness remains low among the general population, resulting in late diagnoses and poor health outcomes. As previous studies have focused on evaluating COPD screening strategies only in high-risk population (e.g., individuals aged >50 years or smokers),^23^^,^^24^ the cost-effectiveness of population-based screening policies in China remains unknown. In this modeling study, our results showed that population-based screening for COPD, using either a one-step or two-step screening method with a screening interval ranging from one-time to annual screening, was cost-effective in China, resulting in ICERs of $8034–13,209/QALY compared with the status quo. Annual two-step screening, using the COPD Screening Questionnaire followed by a portable spirometer test, was the most cost-effective among all screening policies. The cost-effectiveness of COPD screening in China could be further increased by strengthening linkage to care after screening. To our knowledge, this is the first economic evaluation study of population-based screening for COPD in China. Our findings imply that offering COPD screening services in national public health programs is a promising approach to improve health outcomes for individuals living with COPD in China.

To contextualize our study, we compared our results for the status quo scenario with those from several similar modeling studies of COPD in China. Relative to an open-cohort simulation model that projected the health and economic burden of COPD for the population aged 40 years and above in China over 2020–2039,^59^ our model used a higher COPD stage-specific mortality rate and projected higher excess deaths from COPD. On the other hand, our mortality outcomes are better aligned with the overall COPD mortality burden reported by the Global Burden of Disease 2019 study,^10^ as well as mortality outcomes from a comparative modeling study that projected results for a standardized patient profile from seven modeling groups.^60^ In addition, compared with two cost-effectiveness studies that found COPD screening among smokers in China to be cost-effective with an ICER of $1000/QALY^24^ and cost-saving in patients with chronic bronchitis,^23^ our model estimated a higher ICER for the two-step screening policy in the general population. Overall, our model yielded robust cost-effectiveness results, highlighting the potential value of population-based screening for improving COPD care in China.

Our findings provide new insights for population health policymaking related to COPD care. Most international guidelines underscore the importance of early diagnosis of COPD and have primarily focused on case-finding strategies in primary care settings^61^^,^^62^ or screening in pre-determined high-risk subpopulations such as those older than 50 years, smokers, or patients with chronic bronchitis.^23^^,^^49^ These guidelines do not recommend universal screening in asymptomatic individuals,^1^^,^^19^ as no clinical study has demonstrated added health benefits from such programs. However, it is worth noting that the epidemiology of COPD in China is markedly different from that in other countries on which current clinical guidelines are primarily based. In addition to having a much larger population of COPD patients than other countries, COPD is severely underdiagnosed in China. Fewer than 3% of Chinese COPD patients are aware of their condition, which is a much lower figure than what has been observed in other countries (e.g., estimated diagnosis rates are 28% in the US,^63^ 33% in Canada,^64^ and 14–41% in Spain^65^). Implementing systematic screening in the population holds the promise of identifying a substantial number of undiagnosed individuals with COPD in China and ultimately improving their long-term health outcomes through timely intervention. While screening and case-finding strategies among high-risk populations have been shown to improve health outcomes and be cost-effective in China,^23^^,^^24^ they may overlook a significant portion of the COPD population who have few or no risk factors (e.g., nearly half of COPD patients in China are non-smokers^8^).

We considered two screening methods in our study: one-step screening with only a questionnaire and two-step screening with the questionnaire followed by a portable spirometer test. Under the same screening frequency, one-step screening resulted in higher incremental QALY gains at higher costs, as more patients received positive screening results and proceeded to subsequential diagnosis and treatment, compared with two-step screening. Adding a portable spirometer test in the two-step method increases the overall specificity of screening and could save diagnostic test costs for individuals receiving false positives from the initial questionnaire screening. In most cases, two-step screening yielded more favorable cost-effectiveness results than one-step screening. Moreover, the two-step screening method has already been implemented in community-based programs and studies in China, supporting its feasibility in practice.^21^ Importantly, two-step screening could be more practical for residents living in rural communities in China, who may lack adequate and convenient access to COPD diagnostic services because they face long travel distances to the nearest healthcare facility offering COPD diagnostic tests.^66^ More generally, such an approach could be beneficial in resource-limited settings with a significant burden of COPD, although further investigation is needed to confirm this finding and its generalizability to other contexts.

While multiple studies have demonstrated the validity of COPD screening questionnaires in practical settings,^22^^,^^38^^,^^67^ challenges persist when considering the application of these questionnaires to real-world, large-scale, population-based screening programs. The accuracy of screening questionnaires has previously been found to vary by population with different demographic and socioeconomic profiles or from different environments, likely due to the variations in the underlying risk factors of COPD among such different populations.^38^ To address this challenge, researchers have suggested that a pilot study is needed to validate and compare the accuracy of screening instruments first when implementing a screening program in a new population or environment. The cost-effectiveness of screening could then be reassessed to adjust the most cost-effective policy based on the updated estimate of screening accuracy in the given practical context. Further investigation is needed to understand the factors that contribute to disparate accuracy of screening tools, and to inform development of training programs for practitioners to maintain screening quality and thereby achieve optimal outcomes.

For any screening program, improvement in health outcomes relies on subsequent diagnosis and treatment. Our results underline the importance of improving the linkage to care for COPD after screening. We found that higher follow-up diagnosis probability and treatment uptake probability would both substantially increase the cost-effectiveness of screening and lead to better health outcomes. However, the current state of linkage to care for COPD patients in China is far from satisfactory. A study showed that the loss of patients at each COPD care cascade step (screening, diagnosis, treatment, and control)^68^ is much greater than for other chronic diseases, such as hypertension and diabetes, in China.^69^^,^^70^ To maximize the benefits of screening and improve population health, there is an urgent need to substantially improve COPD patients’ access to care.

Financial incentives for COPD management could play a crucial role in improving patients' linkage to diagnosis and treatment. In China, the Basic Public Health Services is a program that provides free disease screening and management to residents at the community level.^43^ While other common chronic diseases such as hypertension and diabetes are already included in the program, COPD is not. Our study demonstrates that screening for COPD can yield comparable or even greater health benefits than screening for other chronic diseases.^71^, ^72^, ^73^ Cost-effectiveness studies on hypertension and diabetes screening reported QALY gains ranging from 0.006 to 0.090 per person,^71^, ^72^, ^73^ whereas our analysis showed QALY gains of 0.018 for the base case and up to 0.060 for an optimistic scenario with substantially improved linkage to care (i.e., with 100% follow-up probability for diagnosis and 80% for treatment). When taking a societal perspective, we expect that COPD screening would become even more cost-effective. This is because the screening could shift the disease burden towards earlier stages, resulting in less morbidity (e.g., hospitalization) and mortality from COPD, which could translate to further reduction in productivity losses stemming from the disease. Therefore, we strongly advocate the inclusion of COPD screening in the Basic Public Health Services program as well as comprehensive coverage of COPD management to enhance patient access to healthcare and improve overall social awareness of COPD.

Our study has several limitations. First, our model did not include the dynamics of individual's smoking status and thus did not capture the potential impact of COPD screening on smoking cessation. This may lead to underestimation of the health benefits of COPD screening policies; however, this would not affect our main cost-effectiveness findings, given that our results already show COPD screening to be robustly cost-effective. Second, the treatment distribution and disease management in our model were based on COPD severity stage, instead of individuals' clinical symptoms, due to the lack of data for supporting model parameterization and validation at the symptom level. However, our extensive sensitivity analysis with respect to treatment distribution demonstrated its limited impact on our cost-effectiveness results. Third, while we utilized as many model parameters (e.g., disease epidemiology) that were specific to China as possible, not all parameters (e.g., disease progression) were derived from the Chinese population. We validated our model by presenting outcomes that were comparable to those of other COPD modeling studies for similar patient profiles. Fourth, we did not explicitly model non-adherence to COPD treatment due to lack of data for quantifying non-adherence patterns and their effects on reduced treatment effectiveness. However, the impact of this limitation on our findings is likely limited, as our sensitivity analysis showed that the cost-effectiveness of COPD screening policies was not sensitive to treatment effectiveness or cost parameters. Lastly, we limited the screening policies in this study to a one-size-fits-all policy structure to generate initial insights about population-based screening policies, without differentiating the policies based on the needs of subpopulations by age group or other risk factors. Future research is needed to evaluate the impact of policies with more flexible structures that can be tailored to at-risk population subgroups.

In conclusion, our findings demonstrate the cost-effectiveness of population-based screening for COPD in China and highlight the value of improving linkage to care for COPD patients. Based on these findings, we strongly recommend that Chinese policymakers consider implementing COPD screening programs and include COPD management in the National Basic Public Health Services, similar to what has been done for other chronic diseases. These measures would likely significantly increase public awareness of COPD, alleviate its disease burden, and improve overall health outcomes for COPD patients in China.

## Contributors

QC, YF, and SC conceptualized and designed the study. YF and SC acquired the data and information for this study. YF and QC conducted data analysis, model implementation, and numerical experiments. YF, QC, KH, and SC interpreted the data and drafted the initial manuscript. KH, WL, PG, TB, TY, and CW critically revised the manuscript. SC, YF, and QC had access to and verified all the data and had final responsibility for the decision to submit the manuscript for publication. All authors participated in preparing the manuscript and approved the final version for submission.

## Data sharing statement

All data supporting the findings of this study are available within the article and its supplementary materials.

## Declaration of interests

All authors have no conflict of interests to declare.
