# Supplementary material for: Cost-effectiveness of population-based screening for chronic obstructive pulmonary disease in China: a simulation modeling study
Source: Lancet Reg Health West Pac. 2024 Apr 29;46:101065. doi: 10.1016/j.lanwpc.2024.101065 (PMC11077022; doi:10.1016/j.lanwpc.2024.101065)
Supplement: Supplementary data [file mmc1.docx]

**Supplementary Materials**

**Cost-effectiveness of population-based screening for Chronic Obstructive Pulmonary Disease in China: a simulation modeling study**

Supplement S1. Detailed Model Description of COPD-SIM

In this section, we describe the model formulation and parameter estimation for the COPD-SIM model in detail. A comprehensive table of model parameters is provided in the next section (**Supplement S2**).

**S1.1 Prevalence and incidence rate**

The number of patients with chronic obstructive pulmonary disease (COPD) in the initial population was determined based on the disease prevalence estimated from the China Pulmonary Health (CPH) study.^1^ The CPH study was a nationally representative cross-sectional study, which included a large sample of 50,991 adults aged 20 years or older and provided the latest estimates of the prevalence of COPD in China. Given the CPH age group-and-sex-stratified COPD prevalence estimates, we computed the initial number of patients with COPD by multiplying prevalence by the number of individuals in each demographic subgroup following the distribution of the general population in China.^2^ To simulate new COPD cases developed within the general population, we utilized estimates of the incidence rate stratified by age group from the Global Burden of Disease (GBD) study.^3^ We then distributed the incidence rate for each sex proportionally to the same sex ratios observed in the prevalence estimates for each age group. For a given sex, we considered the incidence rate to be the same within each age group. In the simulation model, we determined the annual number of incident cases by multiplying the incidence rate by the number of people at that specific age, and then distributed cases evenly to each model period (i.e., three months). The estimated values for prevalence and incidence rates are summarized in **Table S1**.

**Table S1. Age- and sex-specific epidemiological parameters of the simulation cohort.**

|  | **Population (%)^a^** | | **Prevalence (%)** | | **Incidence rate (per 100,000 population)** | |
| --- | --- | --- | --- | --- | --- | --- |
| **Age** | **Male** | **Female** | **Male** | **Female** | **Male** | **Female** |
| 35-39 years | 6.68 | 6.31 | 1.86^b^ | 1.79^b^ | 50.42 | 48.50 |
| 40-49 years | 13.89 | 13.30 | 7.45 | 3.14 | 165.64 | 69.84 |
| 50-59 years | 14.69 | 14.52 | 15.57 | 5.96 | 442.46 | 169.44 |
| 60-69 years | 9.61 | 9.74 | 27.08 | 11.58 | 1053.21 | 450.33 |
| 70-80 years | 5.41 | 5.85 | 42.96 | 20.27 | 5056.24^c^ | 2385.55^c^ |

^a^ In our simulation model, we used age distribution data by single-year age group obtained from the life table of China;^2^ we present the age distribution aggregated by these age groups to be in line with the presentation of prevalence and incidence parameters in this table.

^b^ We used the prevalence estimate for the 20-39-year age group originally reported in the CPH study.^1^

^c^ We used the incidence rate estimate for the 70-85-year age group originally reported in the GBD study.^3^

**S1.2 Disease progression and natural history**

***FEV_1_* and *FEV_1_ %* predicted**: A COPD patient’s lung function was characterized by the forced expiratory volume in one second (*FEV­_1_*), which was tracked over time in our model. A patient’s disease severity was quantified by *FEV_1_ % predicted*, representing the degree of airway obstruction and defined as the *FEV_1_* value divided by the *predicted FEV_1_* value, with the *predicted FEV_1_* values sampled from a normal distribution with mean $\mu$ and standard deviation $\sigma$ calculated from the spirometry predictive equations based on a spirometry-based study in China:^4^

$$log\left( \mu\right)=\beta_{0}+\beta_{1}log\left( \mathrm{age} \right)+ \beta_{2}log\left( \mathrm{height} \right) + M_{spline}(age, sex),$$

$$log\left( \sigma\right)= \beta_{0} + \beta_{1}log\left( \mathrm{age} \right) +S_{spline}(age, sex),$$

where $M_{spline}$ and $S_{spline}$ denote the spline functions for $\mu$ and $\sigma$, respectively. The spline function values for each age and sex combination were reported in the original study^4^. Given the value of *FEV_1_ % predicted*, a patient’s severity stage could be determined according to the GOLD criteria (i.e., *FEV_1_ % predicted* ≥ 80% as GOLD Stage 1, ≥50 and < 80% as GOLD Stage 2, ≥30 and <50% as GOLD Stage 3, and <30% as GOLD Stage 4).^5^

Disease progression was modeled as declines over time in lung function measured in the *FEV_1_* value (**Figure S1**). Baseline *FEV_1_* values for the initial and new COPD patients were initialized by multiplying *FEV_1_ predicted* by *FEV_1_ % predicted*, where the initial *FEV_1_ % predicted* was sampled from the distribution for existing and new COPD patients based on the CPH study.^1^ The *FEV_1_* annual decline rate depended on the GOLD stage, and the GOLD stage was updated after the values of *FEV_1_* and *FEV_1_ % predicted* were recalculated in each cycle. Each patient’s *FEV_1_* decline rate also depended on the occurrence of disease exacerbation and their treatment status in each model cycle.

**Figure S1. Schematic of the natural history simulation model for COPD.** Disease progression was modeled based on the decline in forced expiratory volume in one second (*FEV_1_*) value and dynamic updates of *FEV_1_ % predicted* over time.


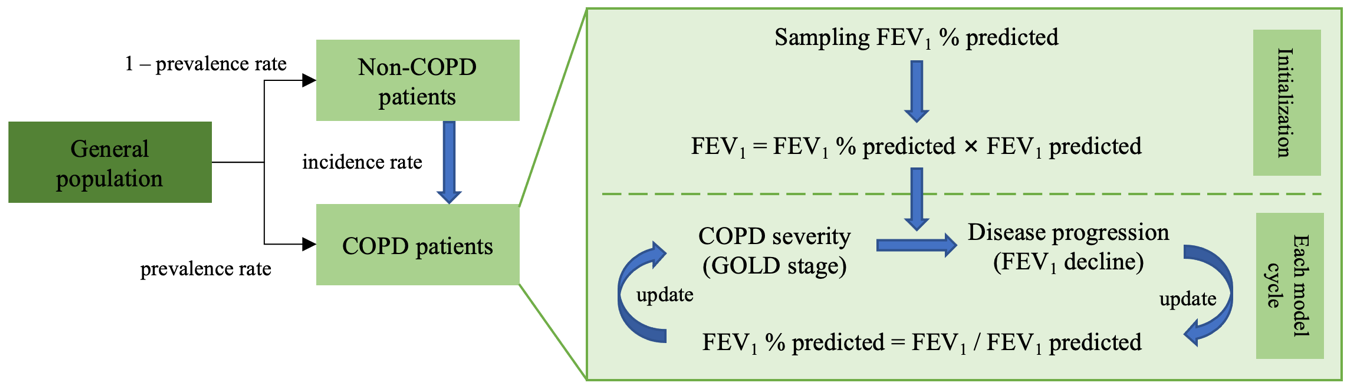


**Exacerbation**: Exacerbation is an important clinical event in the disease course of COPD, marked by increased dyspnea that could reduce quality of life and result in extra costs.^6,7^ Our model considered two types of exacerbation: *non-severe* exacerbation without hospitalization and *severe* exacerbation requiring hospital admission. The frequency of each type differed by COPD severity (i.e., the GOLD stage). An exacerbation event could further accelerate the decline rate of lung function. We estimated that the *FEV_1_* annual decline rate increased by 95.7% after an exacerbation based on results reported from the UPLIFT study.^8^ Since the UPLIFT study only reported the one-year aggregated change in the *FEV_1_* decline rate after exacerbation, data were limited for estimating time-varying effects or projecting long-term effects after exacerbation events; as a result, we conservatively assumed that an exacerbation-induced increase in the *FEV_1_* decline rate would last for 12 months following the latest exacerbation event and remain the same throughout this time period. If another exacerbation occurred during this period, the *FEV_1_* decline rate would remain the same, but the duration of the increased decline rate would be reset—i.e., the patient would continue to experience the increased decline rate for 12 months following the most recent exacerbation.

**COPD-related excess mortality:** Patients with COPD were at a higher risk of mortality. To calculate COPD-related excess mortality, we first adjusted the age- and sex-specific background mortality rates (from the WHO’s life table for China^9^) by the hazard ratios (HRs) for all-cause mortality for each GOLD stage,

$$\boldsymbol{m}_{\boldsymbol{total}}\boldsymbol{=1-}\left( \boldsymbol{1-}\boldsymbol{m}_{\boldsymbol{background}} \right)^{\boldsymbol{HR}}\boldsymbol{,}$$

and then calculated COPD-related excess mortality as $m_{total}-m_{background}$. To estimate the hazard ratios for mortality by GOLD stage, we first reviewed several published studies^10-13^ and found that the estimated hazard ratios varied by study population (**Table S2**). We then selected the estimates that resulted in the closest value for simulated COPD-related deaths to the estimate of COPD-related deaths from the GBD study.^3^ More details on this comparison can be found in the validation results in **Supplement S1.7**.

**Table S2.** **Summary of hazard ratios for all-cause mortality for patients with COPD by GOLD Stage from observational studies.**

| **Study** | **Cohort** | **HR (95% CI) for all-cause mortality** | | | |
| --- | --- | --- | --- | --- | --- |
|  |  | **GOLD Stage 1** | **GOLD Stage 2** | **GOLD Stage 3** | **GOLD Stage 4** |
| Leivseth et al. (2014)^10^ | Included 10,491 adults who participated in the Nord-Trøndelag Health Study (HUNT) in 1995–1997 and were followed through 2009 | Male: 1.23 (0.81-1.85) Female: 1.40 (0.82-2.43) | Male: 1.63 (1.17-2.29) Female: 2.02 (1.39-2.95) | Male: 3.57 (2.60-4.91) Female: 6.50 (4.33-9.75) | Male: 3.57 (2.60-4.91) Female: 6.50 (4.33-9.75) |
| Lan et al. (2017)^11^ | Based on the China Kadoorie Biobank, in which 199,099 men and 287,895 women aged 30-79 years surveyed at baseline were included after excluding those with heart disease, stroke and cancer. | Male: 0.94 (0.82-1.07) Female: 1.05 (0.88-1.26) | Male: 1.01 (0.94-1.09) Female: 1.05 (0.95-1.15) | Male: 1.55 (1.43-1.67) Female: 1.78 (1.60-1.98) | Male: 2.79 (2.50-3.11) Female: 2.80 (2.38-3.29) |
| Mannino et al. (2003)^12^ | A cohort of 5,542 subjects whose pulmonary function was measured as part of the First National Health and Nutrition Examination Survey (NHANES I). | 1.2 (1.01-1.4) | 1.6 (1.4-2.0) | 2.7 (2.1-3.5) | 2.7 (2.1-3.5) |
| Mannino et al. (2006)^13^ | Included 15,759 adult participants, aged 43–66 years at baseline, in the Atherosclerosis Risk in Communities (ARIC) study. | With symptoms: 1.6 (1.2-2.1) Without symptoms: 1.0 (0.8-1.3) | With symptoms: 2.1 (1.7-2.6) Without symptoms: 1.6 (1.3-2.1) | With symptoms: 4.5 (3.5-5.8) Without symptoms: 1.9 (0.97-3.6) | With symptoms: 4.5 (3.5-5.8) Without symptoms: 1.9 (0.97-3.6) |

Abbreviations: HR, hazard ratio; CI, confidence interval; GOLD, the Global Initiative for Chronic Obstructive Lung Disease

S1.3. Diagnosis under the status quo (without screening)

Under the status quo without screening, patients with COPD may still be diagnosed and become aware of their condition through seeking usual care for COPD symptoms or other health conditions. To capture the existing care paths for COPD patients receiving diagnosis without screening, we modeled this process by considering that each patient has a (relatively low) probability of being diagnosed, referred to as self-detection probability, in each period. However, there exist no published studies or data for directly estimating such diagnosis probabilities. Therefore, we employed the *model calibration* approach^14,15^ to determine these parameter values. In particular, we searched for the appropriate values of the diagnosis probabilities by each GOLD stage such that under the status quo, the simulated proportion of COPD patients who were diagnosed at each GOLD stage matched the observed real-world data. Using self-reported data from the CPH study,^16^ we estimated the proportions of COPD patients at GOLD stages 1-4 receiving diagnosis to be 0.63%, 2.0%, 12.9%, and 21.1%, respectively; these figures then served as our calibration targets. The diagnosis probability for a later GOLD stage (e.g., GOLD Stage 4) is unlikely to affect the proportion of patients diagnosed at an earlier GOLD stage (e.g., GOLD Stage 1) because of the irreversible nature of COPD progression; we therefore calibrated the diagnosis probabilities for GOLD Stage 1 to GOLD Stage 4 sequentially. **Figure S2** illustrates how the simulated proportion of patients receiving a diagnosis (by year 10) was affected by the calibration parameter values and how it changed over time relative to the calibration targets. The calibrated parameter value was determined by linear interpolation between the two nearest values of simulation outputs, or by the nearest parameter value if the calibration target was out of the simulation output range.

To account for uncertainty in the calibrated parameter values, we repeated the calibration with 50 independent replications and reported the mean values and their range (**Table S3**). We then validated the (mean) calibrated parameter values in **Figure S3**, which shows that despite some fluctuations, the projected proportions of diagnosed patients remained close to the target values over time, as expected.

**Figure S2. Calibration output for patient’s annual diagnosis probability without screening (an example from one replication of model calibration).** The blue line shows the proportion of diagnosed patients at the end of the tenth year under different values of the annual probability of self-detection. The orange line shows the initial proportion of diagnosed patients.


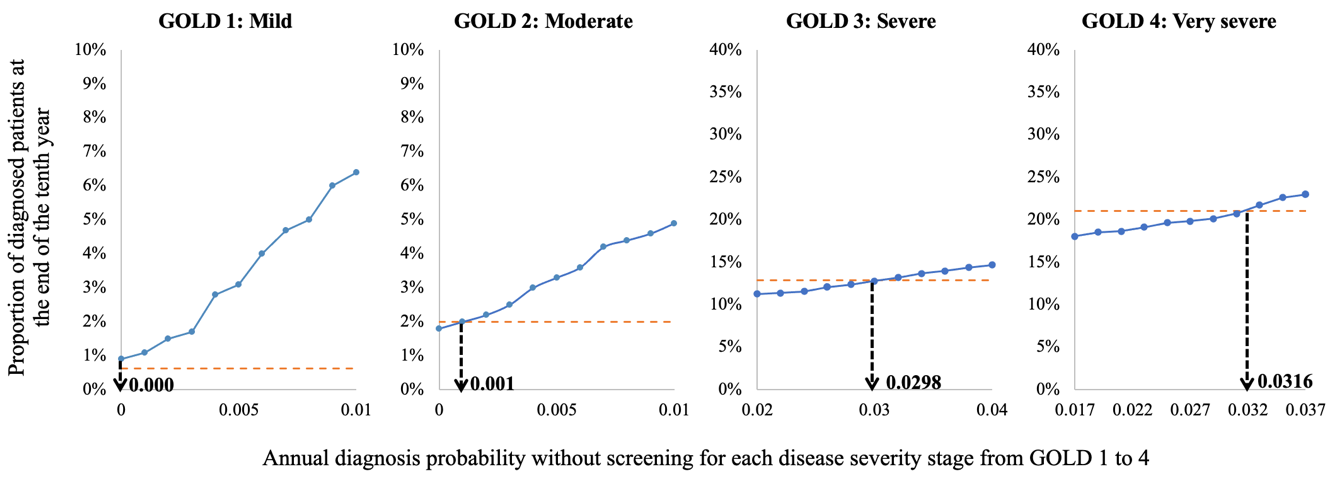


**Table S3. Calibrated values of annual diagnosis probability without screening by GOLD stage.**

| **COPD stage** | **Mean** | **Standard deviation** | **Min** | **Max** |
| --- | --- | --- | --- | --- |
| GOLD Stage 1: Mild | 0.0002 | 0.0004 | 0.0000 | 0.0010 |
| GOLD Stage 2: Moderate | 0.0018 | 0.0005 | 0.0010 | 0.0028 |
| GOLD Stage 3: Severe | 0.0331 | 0.0031 | 0.0214 | 0.0394 |
| GOLD Stage 4: Very severe | 0.0349 | 0.0045 | 0.0281 | 0.0459 |

**Figure S3. The proportion of diagnosed patients by GOLD stage in the first ten years of simulation under the status quo (without screening) with the calibrated model parameters.** The dotted lines represent calibration targets. Despite some fluctuations, the projected values remain close to the target values, which means our model is working as expected with the calibrated parameters.


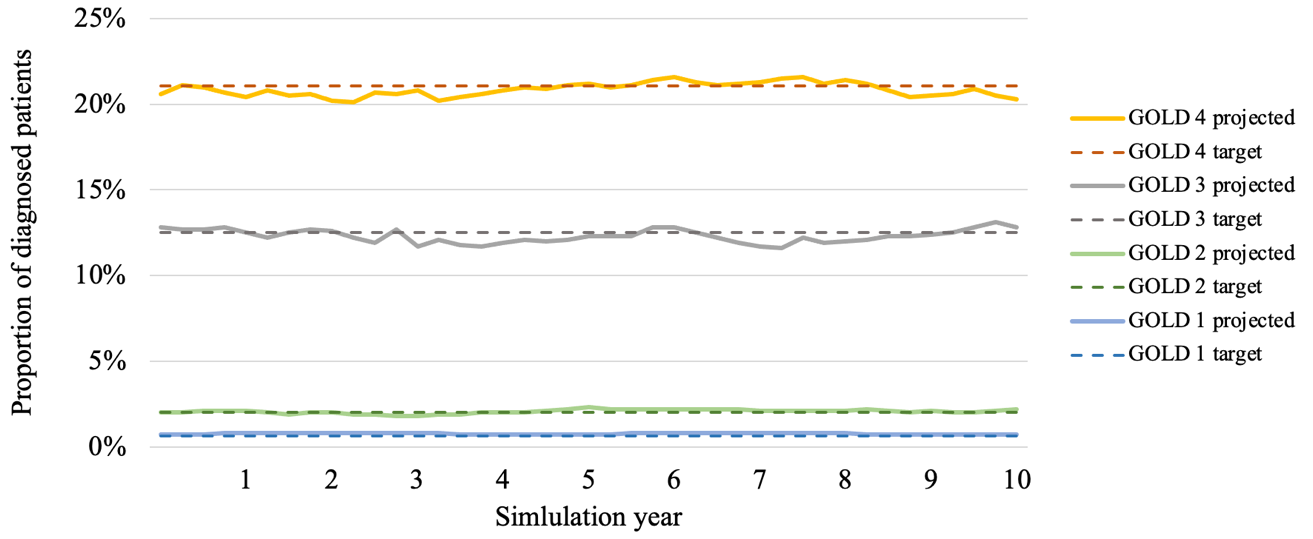


S1.4 Treatment for COPD

Treatment can have a critical impact on the disease course of COPD by reducing the *FEV_1_* decline rate, the exacerbation rate, and the mortality rate.^17,18^ We considered inhaled therapy for COPD, following clinical guidelines for the treatment of stable COPD.^5^ Common medications used for inhaled therapy include short-acting beta2 agonists (SABA), short-acting muscarinic antagonists (SAMA), long-acting muscarinic antagonists (LAMA), long-acting beta2 agonists (LABA), and inhaled corticosteroids (ICS). Short-acting bronchodilators such as SABA and SAMA are usually used as the initial empirical treatment to relieve breathlessness and exercise limitation.^19^ However, there is still a lack of evidence on how short-acting bronchodilators affect lung function over several months or years and how they affect exacerbation rates.^20,21^ Therefore, we assumed that the use of SABA and SAMA would have a limited influence on modeled outcomes over a lifetime horizon and accordingly excluded SABA and SAMA from the treatment methods set in the model. Ultimately, we considered five treatment methods: LABA, LAMA, LABA/ICS, LABA/LAMA, LABA/LAMA/ICS.

Not all diagnosed patients receive treatment. From the CPH study data, we estimated a treatment rate of nearly 30% with long-acting inhaled therapy among diagnosed patients,^16^ and thus assumed that 30% of diagnosed patients received one of the five main types of treatment considered in our simulation. The long-acting therapy treatment rate in China was considerably lower than the estimated rates of 55-65% in developed countries such as the US,^22^ UK,^23^ and Canada.^24^ To further examine the impact of treatment rate on the cost-effectiveness of screening policies, we varied the treatment rate from 20% to 80% in our linkage to care scenario analysis (see the results in *Scenarios of Improved Linkage to Care* section in the main text).

In practice, pharmacological treatments for COPD are primarily based on symptom level (e.g., measured by the modified Medical Research Council dyspnea questionnaire [mMRC], COPD Assessment Test [CAT]) and exacerbation risks in accordance with clinical guidelines.^5^ However, it is challenging to simulate dynamic changes in the symptom level due to lack of the longitudinal empirical data needed for model validation. Thus, we followed convention in COPD modeling literature by simulating the disease dynamics as the changes in *FEV_1_* value and GOLD stage^25,26^ and assign pharmacological treatment based on GOLD stage.^27^ Therefore, we simplified the treatment assignment by evenly distributing treatment options^28^ commonly used for each GOLD stage (**Table S4**). In our sensitivity analysis (**Supplement S1.8**), we relaxed this assumption by experimenting with all 64 (=2*4*4*2) possible combinations of treatment strategies by assigning only one treatment to each GOLD stage. We also tested two treatment scenarios with all patients receiving either the most basic treatment (LABA) or the most advanced triple therapy (LABA/LAMA/ICS) and varied the treatment costs to test the robustness of our results for the overall cost-effectiveness of screening policies.

**Table S4. Treatment distribution by GOLD stage.**

| **Treatment** | **GOLD Stage 1: mild** | **GOLD Stage 2: moderate** | **GOLD Stage 3: severe** | **GOLD Stage 4: very severe** |
| --- | --- | --- | --- | --- |
| LABA | 0.5 | 0.25 |  |  |
| LAMA | 0.5 | 0.25 | 0.25 |  |
| LABA/ICS |  | 0.25 | 0.25 |  |
| LABA/LAMA |  | 0.25 | 0.25 | 0.5 |
| LABA/LAMA/ICS |  |  | 0.25 | 0.5 |

We derived the effects of treatment on the rates of *FEV_1_* decline, COPD exacerbations, and COPD-related mortality in our simulation from several published meta-analyses (**Table S5**). Since these meta-analyses focused on estimating the *relative treatment effectiveness* between different treatments rather than comparing each treatment with the same comparator of no treatment (except for LABA^29^), we derived the effect of each treatment compared with no treatment by aggregating the relative effects between treatments for indirect comparisons, by assuming that the decrease in *FEV_1_* decline rate is additive while the relative risks of exacerbation and mortality are multiplicative (**Table S5**). To minimize the potential bias and uncertainty in the estimation propagated by multiple additions or multiplications, we used the result from studies that included LABA in their treatment comparisons as a common reference whenever available.

**Table S5. Estimated effect of treatment compared with no treatment.**

| **Treatment** | **Comparator** | **Relative effect** | **Cumulative effect compared with no treatment** | **Notes** |
| --- | --- | --- | --- | --- |
| **Decrease in annual *FEV_1_* decline rate (ml)** |  |  |  |  |
| LABA | No treatment^29^ | 73 | 73 |  |
| LAMA | LABA^30^ | 20 | 93 |  |
| LABA/ICS | LABA^30^ | 50 | 123 |  |
| LABA/LAMA | LABA^31^ | 90 | 163 |  |
| LABA/LAMA/ICS | LABA/LAMA^32^ | 38 | 201 |  |
| **Relative risk of exacerbation (moderate/severe)** |  |  |  |  |
| LABA | No treatment^29^ | 0.915 | 0.915 | LABA: 542/1971^a^  No treatment: 600/1997  Relative risk = 0.915 |
| LAMA | LABA^33^ | 0.886 | 0.811 | LAMA: 3169/9935  LABA: 3560/9886  Relative risk = 0.886 |
| LABA/ICS | LABA^30^ | 0.882 | 0.807 | LABA/ICS: 3208/8268  LABA: 3283/7462  Relative risk = 0.882 |
| LABA/LAMA | LABA^31^ | 0.889 | 0.813 | LABA/LAMA: 449/2571  LABA: 648/3297  Relative risk = 0.889 |
| LABA/LAMA/ICS | LABA/LAMA^32^ | 0.700 | 0.622 | Directly reported |
| **Relative risk of mortality** |  |  |  |  |
| LABA | No treatment^29^ | 0.869 | 0.869 | LABA: 254/7214  No treatment: 278/6865 Relative risk = 0.869 |
| LAMA | LABA^30^ | 0.938 | 0.815 | LAMA: 127/11701  LABA: 129/11143  Relative risk = 0.938 |
| LABA/ICS | LABA^30^ | 0.847 | 0.737 | LABA/ICS: 297/10754  LABA: 291/8927  Relative risk = 0.847 |
| LABA/LAMA | LABA^30^ | 1.143 | 0.994 | LABA/LAMA: 31/4126  LABA: 25/3804  Relative risk = 1.143 |
| LABA/LAMA/ICS | LABA/LAMA^34^ | 0.710 | 0.706 | Directly reported |

^a^ For comprehensiveness and transparency, we included the these numbers extracted from the meta-analysis for estimating the crude risk for each treatment and the relative risk between treatments throughout this table.

In our COPD-SIM model, patients continued to receive treatment (with possible changes of therapies) since the treatment was once initiated. Specifically, we considered the effects of treatment on reducing exacerbation and mortality risks as long as the patient received treatment. On the other hand, in consideration of limited data from the literature demonstrating treatment-induced reduction in the annual *FEV_1_* decline rate for periods longer than one year, we assumed in our simulation model that the treatment effect on the *FEV_1_* decline rate lasted for one year, if the patient’s treatment regimen was not changed before then. We also assumed that the reduction in annual *FEV_1_* decline was evenly distributed throughout the year. For example, given that the decrease in the annual *FEV_1_* decline rate with LABA treatment compared with no treatment is 73ml/year, we applied a reduction of 6.08ml (=73ml/12) to the monthly *FEV_1_* decline rate.

When the COPD severity stage (GOLD stage) of a patient currently under treatment changed, we assumed that the patient’s treatment and the reduction in the patient’s *FEV_1_* decline rate would be updated accordingly; however, the *duration* of the treatment-induced reduction in the patient’s *FEV_1_* decline rate would not “reset,” in consideration of the inconclusive evidence from existing clinical data regarding any continuous or long-term effects of treatment on the rate of *FEV_1_* decline.^5^ For example, a patient who started receiving LABA in month one and switched to LABA/ICS in month seven would experience a reduction of 73ml/12 in their *FEV_1_* decline rate over each of the first six months of treatment and a reduction of 123ml/12 over each of months 7-12, but would experience no reduction in their *FEV_1_* decline rate over the remainder of their life. In our sensitivity analysis, we considered an alternative assumption in which the duration of reduction in the *FEV_1_* decline rate reset when a patient’s treatment type changed and evaluated the impact of this alternative assumption on model outcomes.

S1.5. Health utility

Individual health utility was determined based on the baseline utility for the general population in China and adjusted for COPD patients by GOLD stage and at the event of exacerbation as follows:

$$\boldsymbol{Utility=}\boldsymbol{U}_{\boldsymbol{baseline}}\left( \mathbf{age group} \right)\boldsymbol{\times\gamma}\left( \mathbf{GOLD stage} \right)\boldsymbol{\times}\left( \boldsymbol{1-\eta}\left( \mathbf{exacerbation severity} \right) \right)\boldsymbol{.}$$

The baseline utility, $\boldsymbol{U}_{\boldsymbol{baseline}}\left( \mathbf{age group} \right)$, was estimated for age groups of 15-44, 45-64, and ≥65 years from an EQ-5D study conducted in China.^35^ To estimate the multiplier $\boldsymbol{\gamma}\left( \mathbf{GOLD stage} \right)$, we used the health utility estimate for each GOLD stage from a published meta-analysis^36^ and normalized this estimate by the average baseline utility for the population in the age group that was comparable to the mean age of the study samples included in the meta-analysis, following the recommended approach for utilizing health state utilities in cost-effectiveness analysis to account for the effects of comorbidities.^37^ Lastly, we estimated the relative decrease in health utility due to exacerbation, $\boldsymbol{\eta}\left( \mathbf{exacerbation severity} \right)\boldsymbol{,}$ by dividing the disutility from exacerbation by current health utility reported from a clinical study for non-severe (0.103/0.698=14.76%) and severe exacerbation (0.157/0.627=25.04%), respectively.^6^ It is worth noting that although the above-referenced study only included patients at GOLD stages 3-4, we applied the exacerbation-related utility parameters to patients to all stages. In our sensitivity analysis, we evaluated the impact of uncertainty in the exacerbation disutility parameters for patients in GOLD stages 1-2 and those in stages 3-4 separately. The decrease in health utility due to an exacerbation event was assumed to last for one cycle (three months). All parameter value estimates are provided in the comprehensive parameter table in Supplement S2.

S1.6. Cost estimates

We took the payer’s perspective and considered all direct costs of COPD. The cost for each treatment type was obtained from a published study^27^ based on data from the IQVIA Chinese Hospital Pharmaceutical Audit Database. The costs of COPD clinical maintenance, exacerbation, and pneumonia were estimated from published studies conducted across multiple cities in China.^38,39^

To estimate the cost associated with COPD screening policies, we considered both the initial set-up cost for a large public screening program and the variable cost for each screening test. Since no direct estimates of the set-up costs for COPD screening programs exist, we searched for the estimated set-up costs for several other screening programs in China (e.g., cervical cancer,^40^ colorectal cancer,^41,42^ breast cancer,^43^ and lung cancer^44^), which ranged from $0.32 to $3.39 per person for the target population. Therefore, we used a conservative estimate of $4 per person across the entire population (of one million individuals) for the one-time set-up cost of all population-based COPD screening policies in the simulation model.

Variable screening costs include the cost of labor and consumable items (e.g., filters for portable spirometer tests). We estimated the cost of the questionnaire per person screened using the time required for administering the questionnaire multiplied by the wage income per unit of time. The questionnaire was conducted online and required community health workers to give instructions and collect data. We assumed that administering the screening to a single person took a community health worker ten minutes; the average annual income for community health worker was estimated based on the reported average annual income for community service workers in China, which is 60,232 CNY,^2^ equivalent to 30 CNY per hour (or 5 CNY per ten minutes, assuming eight working hours per day and 250 working days per year). The cost of the portable spirometer-based test consisted of the labor cost and consumable item cost (e.g., filters). The cost of hospital-based spirometry testing to diagnose COPD was collected from the Beijing Municipal Medical Insurance Bureau ^45,46^. The estimation of screening costs is summarized in **Table S6**.

**Table S6. Estimation of screening costs.**

| **Cost item** | **Unit cost, CNY (USD)** | **Assumptions and sources** |
| --- | --- | --- |
| Questionnaire | 5 ($0.72) | Labor cost per person screened: 10 min * 0.5 CNY/min = 5.0 CNY |
| Portable spirometer | 20 ($2.90) | Labor cost per person screened: 30min * 0.5 CNY/min = 15 CNY  Consumable item: 5 CNY |
| Spirometry test | 186 ($26.93) | Outpatient fee: 50 CNY  Bronchodilator test: 50 CNY  Chest X-ray: 16 CNY  Chest X-ray photography: 70 CNY |
| Program setup cost | 27.62 ($4) per person for the target population | Other screening programs in China (e.g., cervical cancer,^40^ colorectal cancer,^41,42^ breast cancer,^43^ and lung cancer^44^) |

**S1.7. Model Validation**

In addition to calibrating our model to the distribution of GOLD stages among patients diagnosed with COPD in China, as observed in an epidemiology study^1^ (see more details in Supplement S1.3), we further validated our model with estimates from multiple additional sources.

We first compared our results with those of other COPD modeling studies.^25,47-52^ It is worth noting that most other COPD modeling studies evaluated the cost-effectiveness of treatment and thus focused on patients already diagnosed with COPD. To make our simulation outcomes comparable with the reported disease progression and death outcomes in a previous cross-model comparison study,^53^ we applied our model to the same standardized patient population profile (males aged 65 years and with mean *FEV_1_* of 65% predicted), utilized the same exacerbation frequency and background mortality rate as stated in the reference study,^53^ and reinitialized our simulation. Based on 50 independent replications of simulation runs, our model estimated that 3.6% (range: 2.8%-4.3%) of patients in GOLD Stage 2 progressed to Stage 3 in the initial year, and none (0%-0.1%) of patients in GOLD Stage 2 progressed to Stage 4. These simulation outcomes are well aligned with the ranges reported in other modeling studies (Table S7). For the same initial patient population, our model projected an overall mortality rate of 2.5% (2.3%-2.8%) and a COPD-related mortality rate of 1.0% (0.9%-1.4%), which were also comparable with other studies.

We also compared the projected total COPD-related mortality burden in the general population with estimates from the GBD 2019 study.^3^ We aggregated the age-specific estimates of COPD-related deaths from the GBD 2019 data for adults aged 35-80 years to be consistent with the age range of the initial cohort in our simulation model. Our model projected an overall COPD-related mortality rate of 81.7 (72.0-92.1) per 100,000 population, which was close to the rate of 70.9 per 10,000 population estimated from the GBD data.

Table S7. Comparison of model results with estimates from other sources for model validation.

| Measure | Model output | Estimates from other sources | Sources |
| --- | --- | --- | --- |
| Mortality |  |  |  |
| Probability of death in the first year for a 65-year-old male at GOLD Stage 2 (moderate COPD, mean *FEV_1_* of 65% predicted) without treatment |  |  |  |
| From all-causes | 2.5% (2.3%-2.8%) | 2.7%-7.4% | Cross-model comparison study^53^ |
| From COPD | 1.0% (0.9%-1.4%) | 1.2%-5.9%^a^ | Cross-model comparison study^53^ |
| Mortality rate in the population of adults aged 35-80 years (per 100,000) | 81.7 (72.0-92.1) | 70.9 (60.7-86.5)^b^ | GBD 2019 data^3^ |
| Transition probability in the first year for a 65-year-old male at GOLD Stage 2 (moderate COPD) |  |  |  |
| Stage 2 to Stage 3 | 3.6% (2.8%-4.3%) | 1.1%-8.3%^c^ | Cross-model comparison study^53^ |
| Stage 2 to Stage 4 | 0% (0%-0.1%) | 0%-0.4%^c^ |  |

^a^ Calculated as all-cause mortality minus non-COPD-related mortality.

^b^ Since the mortality rate for the 35-80-year age group was not directly available from the 2019 GBD study,^3^ we approximated the rate by summing the number of COPD-related deaths in each five-year age group and then dividing the total by the population aged 35-80 years in China.^2^ To approximate the estimation uncertainty, we started with the reported mortality rate of 92.4 (95% CI: 79.2-112.7) per 100,000 population for the ≥20-year age group (the closest to the 35-80-year age group in our simulation), and then scaled its confidence interval proportionally based on the relative scale of the point estimate values (i.e., from 92.4 to 70.9 per 100,000).

^c^ The cited ranges exclude one study,^50^ which reported a 32% probability of transitioning from Stage 2 to Stage 3 and a 6.6% probability of transitioning from Stage 2 to Stage 4, as these figures were much higher than those generated by the other six reviewed models. This might be explained by the fact that the patients with moderate COPD in this study population fell mostly at the severe end of the lung function range for moderate COPD.

Lastly, we validated our model with the predicted lung function decline for COPD based on the Lung Health Study (LHS) by Zafari and colleagues.^54^ They derived an individualized time-series equation for predicting the trajectory of *FEV_1_* and validated it in external datasets. They also developed a web-based lung function prediction tool^55^ that predicts the *FEV_1_* value for up to 11 years based on individual clinical variables. Using this tool, we compared the tool’s prediction with the *FEV_1_* trajectory simulated from our model (**Figure S4**). We first used the tool’s basic model (based on baseline *FEV_1_* value only) and compared it with our simulation model by setting the same baseline *FEV_1_* (**Figure S4-A**). Next, to compare FEV_1_ trajectory by each sex, the tool predicted the *FEV_1_* trajectories for current smokers and sustained quitters separately, whereas our model only considered the general COPD population in China without differentiating by smoking status. For meaningful comparisons, we used the tool’s predicted *FEV_1_* trajectories for current smokers and sustained quitters as the lower and upper limits, respectively, of the *FEV_1_* values (while setting other baseline characteristics at the average level of the simulation cohort, which was in line with the CPH study cohort^16^). We then examined whether the *FEV_1_* trajectories simulated from our model were within these ranges (**Figure S4-(B) and (C)**). In **Figure S4**, the blue solid and dotted lines represent the average and the range from our simulation model, and the orange lines represent the prediction results from Zafari’s model. These results demonstrate that our simulation projections are well in line with the predictions from Zafari’s study.

**Figure S4**. Comparison of predicted *FEV_1_* trajectory from COPD-SIM and Zafari’s study for (A) baseline patient characteristics with age of 55 years and initial *FEV_1_* = 2.75L, (B) average female patient in the COPD-SIM with age of 62 years and initial *FEV_1_*=1.66L, and (C) average male patient in the COPD-SIM with age of 61 years and initial *FEV_1_*=2.19L.


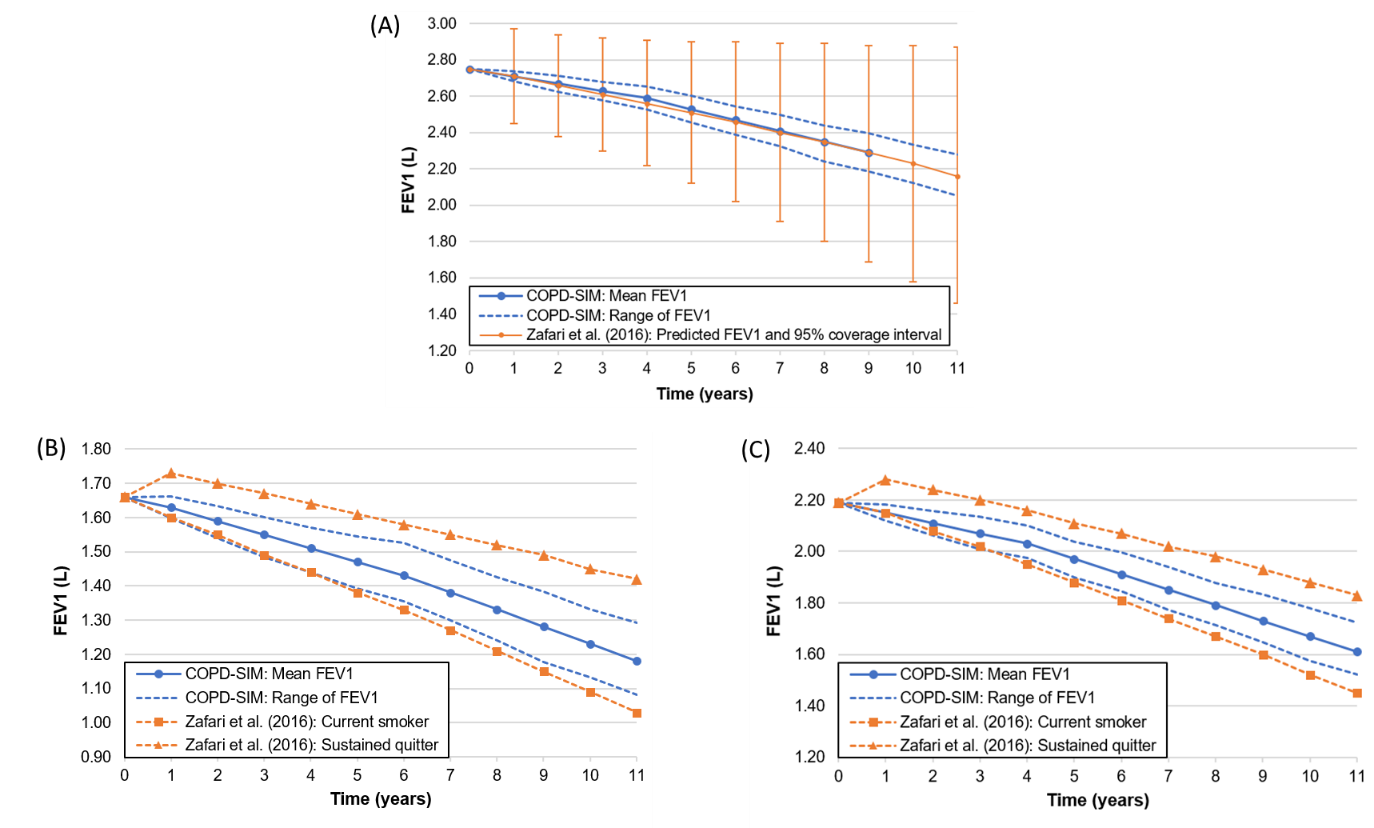


S1.8 Quantifying Uncertainty of Model Parameters

We conducted comprehensive sensitivity analyses to assess the impact of input parameter uncertainty on model outcomes.

S1.8.1 One-way sensitivity analysis

In the one-way sensitivity analysis, we used the reported 95% confidence interval for the point estimate's range whenever available from the published studies; otherwise, we set the range as ±20% of the point estimate (i.e., the baseline value). For some parameters that were stratified by subgroups (e.g., background mortality by age, prevalence and incidence by age and sex, and *FEV_1_* annual decline rate by GOLD stages), we applied a multiplier to the parameter values across all subgroups to ensure “synchronized” changes relative to the uncertainty range of each individual subgroup. For example, when considering higher hazard ratios for the mortality by GOLD stage, we increase the hazard ratio of each GOLD stage by the same percentage towards the upper limit of its own uncertainty range.

S1.8.2 Additional scenario analysis for key screening and treatment parameters

One potential challenge of applying population-base COPD screening in practice is the varying accuracy of screening tests in the general population. To further explore the impact of variations in screening test validity, we re-evaluated the COPD screening policies and their cost-effectiveness under different sensitivity and specificity parameters for COPD-SQ^56-59^ and different types of portable spirometers^60^ that have been reported in the literature (Table S13). Considering the generalizability of the proposed COPD screening methods to other practical screening questionnaires, such as COPD-PS^59,61-63^ and COPD-CPHS,^64^ we also included the screening sensitivity and specificity of these additional screening tools in our analysis (Table S13).

While our model followed a common COPD modeling framework that used GOLD stage to inform treatment strategies for simplification, COPD treatment and management in practice are mainly based on symptom levels and exacerbations. To address this model limitation, we varied the treatment strategies by assigning a different treatment to each GOLD stage. We also tested the cases assigning either the most basic treatment (LABA) or the most advanced triple therapy (LABA/LAMA/ICS) for all patients, respectively (Table S14). To account for the cost implications of different treatment strategies, we included additional scenarios with different COPD treatment costs and examined their impacts on the cost-effectiveness results (Table S15).

S1.8.3 Probabilistic sensitivity analysis (PSA)

In the probabilistic sensitivity analyses, for individual parameters, we drew samples of parameter values following the recommended statistical distributions based on the parameter type and the method for determining the parameters of the sampling distributions.^65,66^ Specifically, we drew from gamma distributions for the rate and cost parameters; beta distributions for the probabilities, sensitivity, specificity, and utility parameters (with values between 0 and 1); and log-normal distributions for the relative risk and hazard ratio parameters. For group parameters, we sampled the value of the multiplier from a gamma distribution and applied the same multiplier value to the parameters within the same group. We applied the Method of Moments to estimate the parameters for each distribution. That is, we back calculated the distribution parameters by assuming the base case value as the mean and the half-width of the uncertainty interval as two times the standard deviation. All detailed parameter ranges and sampling distributions can be found in Supplement S2 Table S8.

Supplement S2. Additional Details of Model Parameters

**Table S8. Summary of key model parameters.**

| **Parameter** | **Base case value** | **Range** | **Sampling Distribution** | **Source** |
| --- | --- | --- | --- | --- |
| ***Population and COPD epidemiology parameters*** |  |  |  |  |
| Age and sex distribution | **Table S9** |  |  | ^2^ |
| Age- and sex-specific prevalence rate of COPD | **Table S1** | ±20% | Gamma(100, 0.01)^a, b^ | ^1^ |
| Age- and sex-specific incidence rate of COPD | **Table S1** | ±20% | Gamma(100, 0.01)^a^ | ^1,3^ |
| Age and sex-specific background mortality | Life table | ±20% | Gamma(100, 0.01)^a^ | ^9^ |
| Height distribution for COPD patients, normally distributed |  |  |  | ^1^ |
| Male | $\mu$=166.22, $\sigma$=6.97 |  |  |  |
| Female | $\mu$=155.56, $\sigma$=6.45 |  |  |  |
| Diagnosis rate in the initial COPD cohort (%) |  | ±20% |  | ^16^ |
| GOLD Stage 1: mild | 0.63 | (0.5, 0.76) | Gamma(100, 0.0063) |  |
| GOLD Stage 2: moderate | 2.00 | (1.6, 2.4) | Gamma(100, 0.02) |  |
| GOLD Stage 3: severe | 12.87 | (10.3, 15.44) | Gamma(100, 0.1287) |  |
| GOLD Stage 4: very severe | 21.04 | (16.83, 25.25) | Gamma(100, 0.2104) |  |
| ***Disease Progression and Natural History*** |  |  |  |  |
| Distribution of *FEV_1_* % predicted for the initial population, normally distributed |  | ±20% ^c^ | Gamma(100, 0.01)^a^ | ^16^ |
| Male | $\mu$=0.797, $\sigma$=0.206 |  |  |  |
| Female | $\mu$=0.835, $\sigma$=0.206 |  |  |  |
| Distribution of *FEV_1_* % predicted for the incidence, normally distributed |  | ±20% ^c^ | Gamma(100, 0.01)^a^ | ^16^ |
| Male | $\mu$=1.014, $\sigma$=0.906 |  |  |  |
| Female | $\mu$=1.034, $\sigma$=0.107 |  |  |  |
| *FEV_1_* annual decline (ml) (normally distributed) |  | ±20% ^c^ | Gamma(100, 0.01)^a^ | ^67,68^ |
| GOLD Stage 1: mild | $\mu$=40, $\sigma$=5 ^d^ |  |  |  |
| GOLD Stage 2: moderate | $\mu$=60, $\sigma$=5 |  |  |  |
| GOLD Stage 3: severe | $\mu$=56, $\sigma$=5 |  |  |  |
| GOLD Stage 4: very severe | $\mu$=34, $\sigma$=5 |  |  |  |
| Non-severe exacerbation: Annual rate ^e^ |  | ±20% |  | ^69^ |
| GOLD Stage 1: mild | 0.71 | (0.57, 0.85) | Gamma(100, 0.007) |  |
| GOLD Stage 2: moderate | 1.01 | (0.81, 1.21) | Gamma(100, 0.01) |  |
| GOLD Stage 3: severe | 1.39 | (1.11, 1.67) | Gamma(100, 0.014) |  |
| GOLD Stage 4: very severe | 1.82 | (1.46, 2.18) | Gamma(100, 0.018) |  |
| Severe exacerbation: Annual rate ^e^ |  |  |  | ^69^ |
| GOLD Stage 1: mild | 0.11 | (0.02, 0.56) | Gamma(0.66, 0.166) |  |
| GOLD Stage 2: moderate | 0.16 | (0.07, 0.33) | Gamma(6.06, 0.026) |  |
| GOLD Stage 3: severe | 0.22 | (0.20, 0.23) | Gamma(860.44, 0) |  |
| GOLD Stage 4: very severe | 0.28 | (0.14, 0.63) | Gamma(5.22, 0.054) |  |
| Increase of lung function decline rate after an exacerbation | 95.7% | (76.6, 114.8) | Gamma(100, 0.957) | ^8^ |
| Pneumonia: Annual rate ^e^ | 0.00224 | (0.002, 0.003) | Gamma(100, 0) | ^70^ |
| Annual probability of self-detection due to COPD symptoms |  |  |  | Calibrated (S1.3) |
| GOLD Stage 1: mild | 0.0002 | (0, 0.001) | Beta(0.64, 3198) ^f^ |  |
| GOLD Stage 2: moderate | 0.0018 | (0.001, 0.003) | Beta(15.97, 8856) |  |
| GOLD Stage 3: severe | 0.0331 | (0.021, 0.039) | Beta(52.28, 1527) |  |
| GOLD Stage 4: very severe | 0.0349 | (0.028, 0.046) | Beta(59.33, 1641) |  |
| Hazard ratio of mortality for COPD patients |  |  |  | ^12^ |
| GOLD Stage 1: mild | 1.2 | (1, 1.6) | Log-normal(0.17, 0.02) ^g^ |  |
| GOLD Stage 2: moderate | 1.6 | (1, 2.1) | Log-normal(0.46, 0.03) |  |
| GOLD Stage 3: severe | 2.7 | (1.6, 6.5) | Log-normal(0.9, 0.19) |  |
| GOLD Stage 4: very severe | 2.7 | (1.9, 6.5) | Log-normal(0.91, 0.17) |  |
| ***Screening*** |  |  |  |  |
| COPD screening questionnaire (COPD-SQ) ^h^ |  |  |  | ^56^ |
| Sensitivity | 0.57 | (0.51, 0.63) | Beta(171.43, 129) |  |
| Specificity | 0.82 | (0.74, 0.9) | Beta(71.18, 16) |  |
| Portable spirometer ^h^ |  |  |  | ^60^ |
| Sensitivity | 0.85 | (0.81, 0.88) | Beta(353.03, 62) |  |
| Specificity | 0.85 | (0.75, 0.94) | Beta(47.18, 8) |  |
| ***Treatment*** |  |  |  |  |
| Decrease in *FEV_1_* decline rate (ml, 12 months in total) |  | ±20% |  | S1.4 |
| LABA | 73 | (58.4, 87.6) | Gamma(100, 0.73) | ^29^ |
| LAMA | 93 | (74.4, 111.6) | Gamma(100, 0.93) | ^33^ |
| LABA/ICS | 123 | (98.4, 147.6) | Gamma(100, 1.23) | ^30^ |
| LABA/LAMA | 163 | (130.4, 195.6) | Gamma(100, 1.63) | ^31^ |
| LABA/LAMA/ICS | 201 | (160.8, 241.2) | Gamma(100, 2.01) | ^32^ |
| Relative risk of exacerbation |  | ±20% |  | S1.4 |
| LABA | 0.915 | (0.732, 1) | Log-normal(-0.09, 0.01) | ^29^ |
| LAMA | 0.811 | (0.649, 0.973) | Log-normal(-0.21, 0.01) | ^33^ |
| LABA/ICS | 0.807 | (0.646, 0.968) | Log-normal(-0.22, 0.01) | ^30^ |
| LABA/LAMA | 0.813 | (0.65, 0.976) | Log-normal(-0.21, 0.01) | ^31^ |
| LABA/LAMA/ICS | 0.622 | (0.498, 0.746) | Log-normal(-0.48, 0.01) | ^32^ |
| Relative risk of mortality |  | ±20% |  | S1.4 |
| LABA | 0.869 | (0.695, 1) | Log-normal(-0.14, 0.01) | ^29^ |
| LAMA | 0.815 | (0.652, 0.978) | Log-normal(-0.21, 0.01) | ^30^ |
| LABA/ICS | 0.737 | (0.59, 0.884) | Log-normal(-0.31, 0.01) | ^30^ |
| LABA/LAMA | 0.994 | (0.795, 1) | Log-normal(-0.01, 0.003) | ^30^ |
| LABA/LAMA/ICS | 0.706 | (0.565, 0.847) | Log-normal(-0.35, 0.01) | ^34^ |
| Relative risk of pneumonia from treatment including ICS | 1.6 | (1.33, 1.92) | Log-normal(0.47, 0.01) | ^71^ |
| ***Health-related quality of life*** |  |  |  |  |
| Quality of life for general population ^h^ |  |  |  | ^35^ |
| Ages 20-44 years | 0.991 | (0.977, 0.992) | Beta(627.54, 5.7) |  |
| Ages 45-64 years | 0.975 | (0.946, 0.981) | Beta(309.43, 7.93) |  |
| Ages >65 years | 0.905 | (0.795, 0.916) | Beta(84.12, 8.83) |  |
| Utility multiplier for COPD ^h^ |  |  |  | ^35,36^ |
| GOLD Stage 1: mild | 0.857 | (0.795, 0.921) | Beta(104, 17.35) |  |
| GOLD Stage 2: moderate | 0.816 | (0.787, 0.846) | Beta(571.78, 128.93) |  |
| GOLD Stage 3: severe | 0.749 | (0.713, 0.786) | Beta(417.38, 139.87) |  |
| GOLD Stage 4: very severe | 0.655 | (0.591, 0.719) | Beta(144.66, 76.2) |  |
| Relative decrease in health utility for a patient undergoing exacerbation ^i^ |  |  |  | ^8^ |
| Non-severe exacerbation: GOLD Stages 1-2 ^j^ | 14.76% | (0, 0.421) | Beta(1.51, 8.79) |  |
| Non-severe exacerbation: GOLD Stages 3-4 | 14.76% | (0, 0.421) | Beta(1.51, 8.79) |  |
| Severe exacerbation: GOLD Stages 1-2 ^j^ | 25.04% | (0, 0.621) | Beta(1.7, 5.09) |  |
| Severe exacerbation: GOLD Stages 3-4 | 25.04% | (0, 0.621) | Beta(1.7, 5.09) |  |
| ***Cost (US$)*** |  |  |  |  |
| Screening and diagnostic cost (per person) |  | ±20% |  | S1.6 |
| Questionnaire | 0.72 | (0.58, 0.87) | Gamma(100, 0.01) |  |
| Portable spirometer | 2.90 | (2.32, 3.47) | Gamma(100, 0.03) |  |
| Diagnostic spirometry test | 26.93 | (21.54, 32.32) | Gamma(100, 0.27) |  |
| Program setup cost | 4.00 | (3.2, 4.8) | Gamma(100, 0.04) |  |
| Monthly maintenance cost (per person) |  | ±20% |  | ^38^ |
| GOLD Stage 1: mild | 7.66 | (6.13, 9.2) | Gamma(100, 0.08) |  |
| GOLD Stage 2: moderate | 24.25 | (19.4, 29.1) | Gamma(100, 0.24) |  |
| GOLD Stage 3: severe | 34.56 | (27.65, 41.47) | Gamma(100, 0.35) |  |
| GOLD Stage 4: very severe | 54.04 | (43.23, 64.84) | Gamma(100, 0.54) |  |
| Monthly treatment cost (per person) |  | ±20% |  | ^27^ |
| LABA | 20.21 | (16.17, 24.26) | Gamma(100, 0.2) |  |
| LAMA | 41.17 | (32.94, 49.41) | Gamma(100, 0.41) |  |
| LABA/ICS | 18.50 | (14.8, 22.2) | Gamma(100, 0.19) |  |
| LABA/LAMA | 104.58 | (83.67, 125.5) | Gamma(100, 1.05) |  |
| LABA/LAMA/ICS | 59.68 | (47.74, 71.61) | Gamma(100, 0.6) |  |
| Exacerbation cost (per event) |  | ±20% |  | ^38^ |
| Non-severe | 68.24 | (54.59, 81.89) | Gamma(100, 0.68) |  |
| Severe | 2987.06 | (2389.65, 3584.47) | Gamma(100, 29.87) |  |
| Pneumonia cost | 2039.44 | (1631.55, 2447.33) | Gamma(100, 20.39) | ^39^ |

^a^ Sampling distribution for the multiplier applied to the given group of model parameters.

^b^ Parameters of distribution Gamma($\alpha$, $\beta$) were estimated by $\alpha=\mu^{2}/\sigma^{2}$ and $\beta=\sigma^{2}/\mu$, assuming the base case value as the mean $\mu$ and half-width of the uncertainty range as two times of the standard deviation $\sigma$.

^c^ Scaling factor applied to the sampled value for each patient.

^d^ The value of $\sigma$ for the sampling distribution was based on the largest standard error of FEV_1_ annual decline rate by GOLD Stages as reported in UPLIFT study.^72^

^e^ Rates are converted to probability under an exponential assumption using the formula $p=1-e^{-rt}$, where t denotes cycle length (1/4 year).

^f^ Parameters of distribution Beta($\alpha$, $\beta$) were estimated by $\alpha=\mu\times(\mu\times(1-\mu)/\sigma^{2}-1)$ and $\beta=\alpha/\mu-\alpha$, assuming the base case value as the mean $\mu$ and half-width of the uncertainty range as two times of the standard deviation $\sigma$.

^g^ Parameters of distribution log-normal($\mu$, $\sigma^{2}$) were estimated by $\mu=\ln\left( a \right)-ln(1+b^{2}/a^{2})/2$ and $\sigma^{2}=ln(1+b^{2}/a^{2})$, assuming the base case value as the mean $a$ and half-width of the uncertainty range as two times of the standard deviation $b$.

^h^ Truncated at one if the value after scaling is greater than one.

^i^ To estimate these ranges, we used the reported disutility of 0.103 (SD=0.191) for non-severe exacerbation and 0.157 (SD=0.245) for severe exacerbation,^6^ considered the range specified by mean±SD (with the lower limit truncated by 0), and scaled the value by the reported current health value of 0.698 and 0.627^6^ to obtain the ranges of the relative utility decrease for non-severe and severe exacerbations, respectively.

^j^ We used the same values for GOLD Stages 1-2 and GOLD Stages 3-4 in the base case, but these parameters were changed independently in the sensitivity analysis.

*Abbreviations:* COPD, chronic obstructive pulmonary disease; GOLD, the Global Initiative for Chronic Obstructive Lung Disease; *FEV_1_*, forced expiratory volume in one second; LABA, long-acting beta_2_-agonists; LAMA, long-acting muscarinic antagonists; ICS, inhaled corticosteroids.

**Table S9. Age and sex distribution of the general population in China.**

| **Age** | **Male (%)** | **Female (%)** | **Total (%)** |
| --- | --- | --- | --- |
| 35 years | 1.32 | 1.25 | 2.57 |
| 36 years | 1.32 | 1.24 | 2.56 |
| 37 years | 1.29 | 1.21 | 2.50 |
| 38 years | 1.47 | 1.39 | 2.86 |
| 39 years | 1.28 | 1.22 | 2.50 |
| 40 years | 1.18 | 1.12 | 2.30 |
| 41 years | 1.30 | 1.23 | 2.54 |
| 42 years | 1.25 | 1.19 | 2.44 |
| 43 years | 1.19 | 1.14 | 2.33 |
| 44 years | 1.33 | 1.27 | 2.59 |
| 45 years | 1.36 | 1.30 | 2.66 |
| 46 years | 1.48 | 1.43 | 2.91 |
| 47 years | 1.55 | 1.49 | 3.05 |
| 48 years | 1.59 | 1.54 | 3.13 |
| 49 years | 1.65 | 1.59 | 3.24 |
| 50 years | 1.73 | 1.69 | 3.42 |
| 51 years | 1.62 | 1.59 | 3.21 |
| 52 years | 1.73 | 1.70 | 3.43 |
| 53 years | 1.38 | 1.37 | 2.75 |
| 54 years | 1.57 | 1.53 | 3.10 |
| 55 years | 1.53 | 1.50 | 3.03 |
| 56 years | 1.48 | 1.48 | 2.96 |
| 57 years | 1.72 | 1.69 | 3.42 |
| 58 years | 1.26 | 1.26 | 2.51 |
| 59 years | 0.68 | 0.71 | 1.39 |
| 60 years | 0.85 | 0.85 | 1.69 |
| 61 years | 0.81 | 0.79 | 1.59 |
| 62 years | 1.03 | 1.00 | 2.03 |
| 63 years | 1.14 | 1.12 | 2.26 |
| 64 years | 1.02 | 1.03 | 2.05 |
| 65 years | 1.06 | 1.08 | 2.14 |
| 66 years | 1.05 | 1.08 | 2.13 |
| 67 years | 0.94 | 0.98 | 1.92 |
| 68 years | 0.93 | 0.98 | 1.91 |
| 69 years | 0.78 | 0.83 | 1.61 |
| 70 years | 0.74 | 0.78 | 1.52 |
| 71 years | 0.73 | 0.75 | 1.47 |
| 72 years | 0.61 | 0.64 | 1.25 |
| 73 years | 0.58 | 0.61 | 1.19 |
| 74 years | 0.51 | 0.56 | 1.07 |
| 75 years | 0.45 | 0.49 | 0.94 |
| 76 years | 0.43 | 0.47 | 0.90 |
| 77 years | 0.37 | 0.42 | 0.79 |
| 78 years | 0.35 | 0.40 | 0.74 |
| 79 years | 0.34 | 0.39 | 0.73 |
| 80 years | 0.30 | 0.35 | 0.65 |
| Total | 50.28 | 49.72 | 100.00 |

Source: China Statistical Yearbook 2021 ^2^. The distribution was rescaled between ages 35-80 years.

Supplement S3. Additional Results of Simulation Modeling Analysis

**Figure S5. Projected cost by category for all screening policies in a cohort of one million individuals over a lifetime horizon.**

**
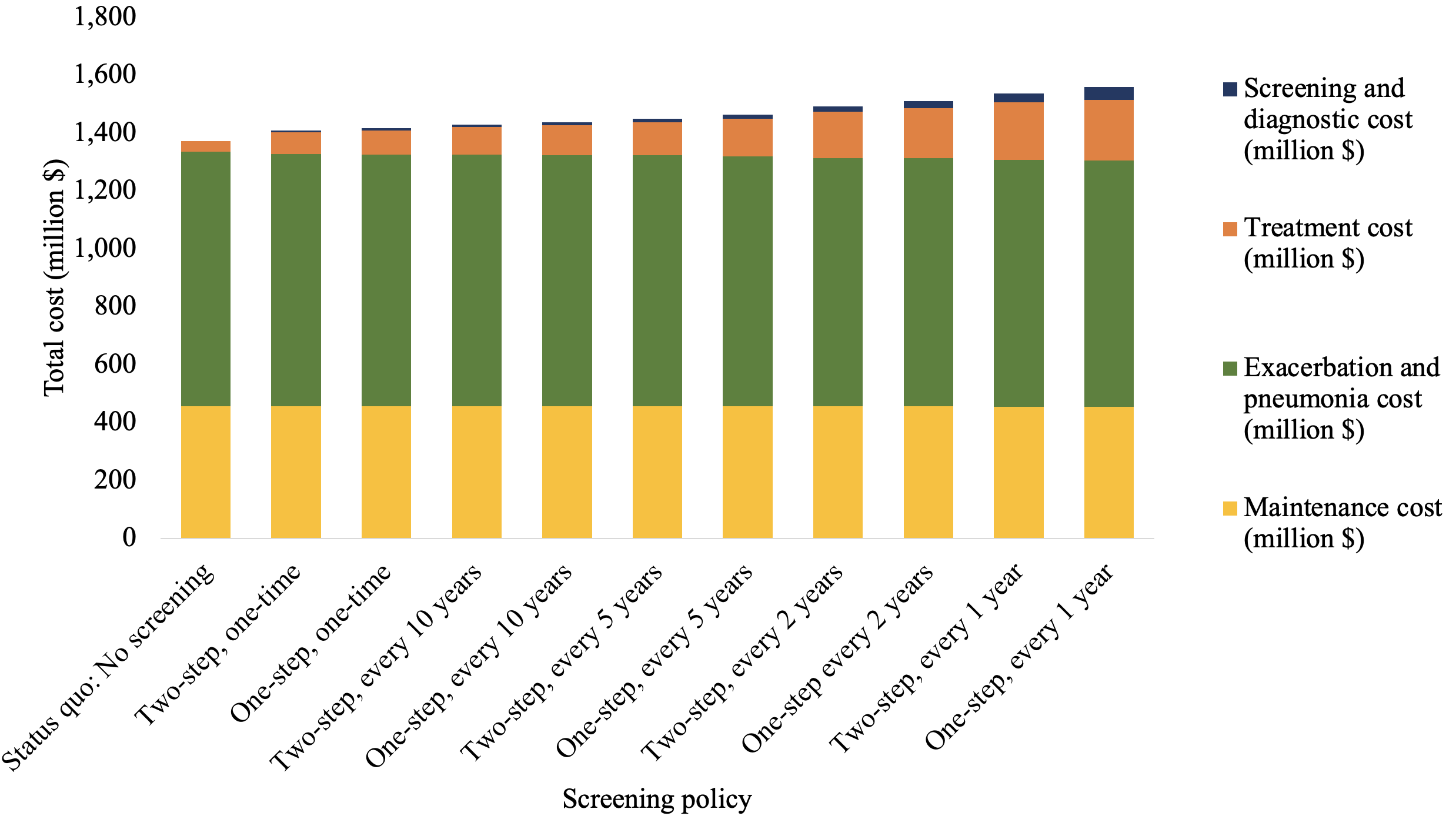
**

**Figure S6. The most cost-effective screening policy and incremental cost-effectiveness ratio (ICER) compared with the status quo under different probabilities of follow-up diagnosis with a fixed probability of treatment uptake of 30%.**


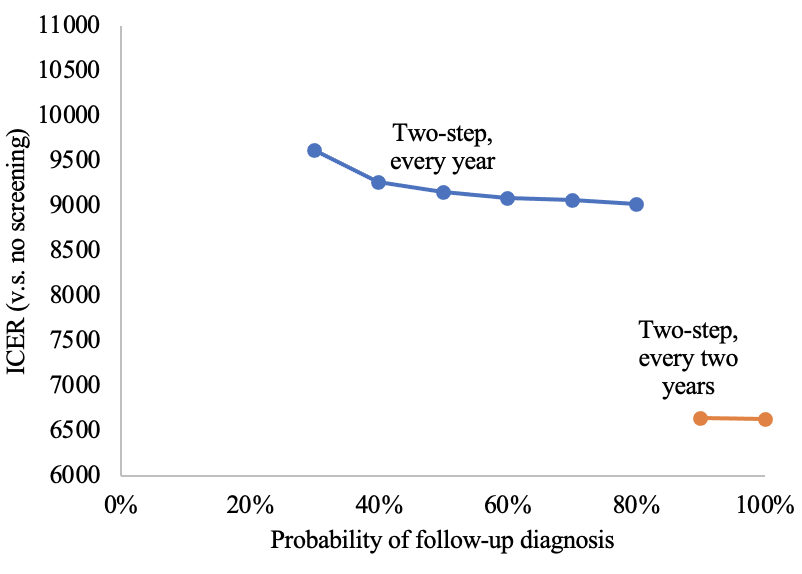


**Figure S7. The most cost-effective screening policy and incremental cost-effectiveness ratio (ICER) compared with the status quo under different probabilities of treatment uptake with a fixed probability of follow-up diagnosis of 40%.**


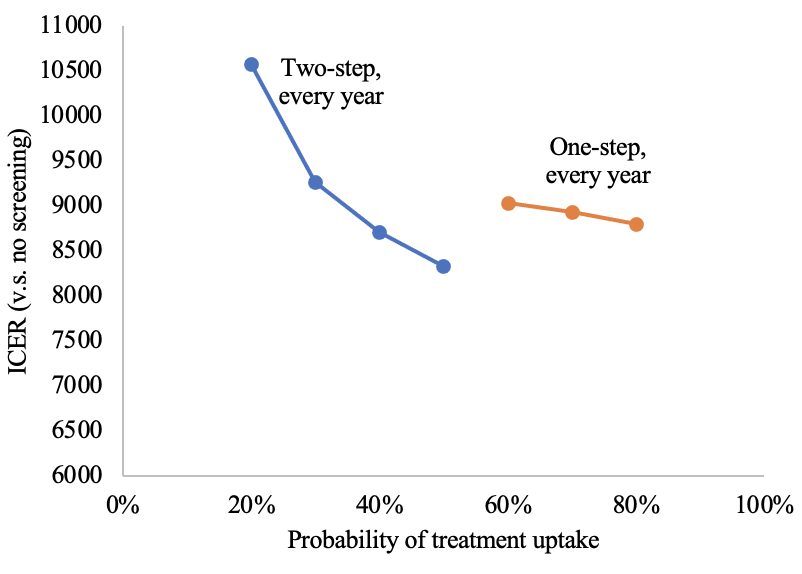


Figure S8. Tornado diagram for one-way deterministic sensitivity analysis: two-step screening every year vs. status quo.


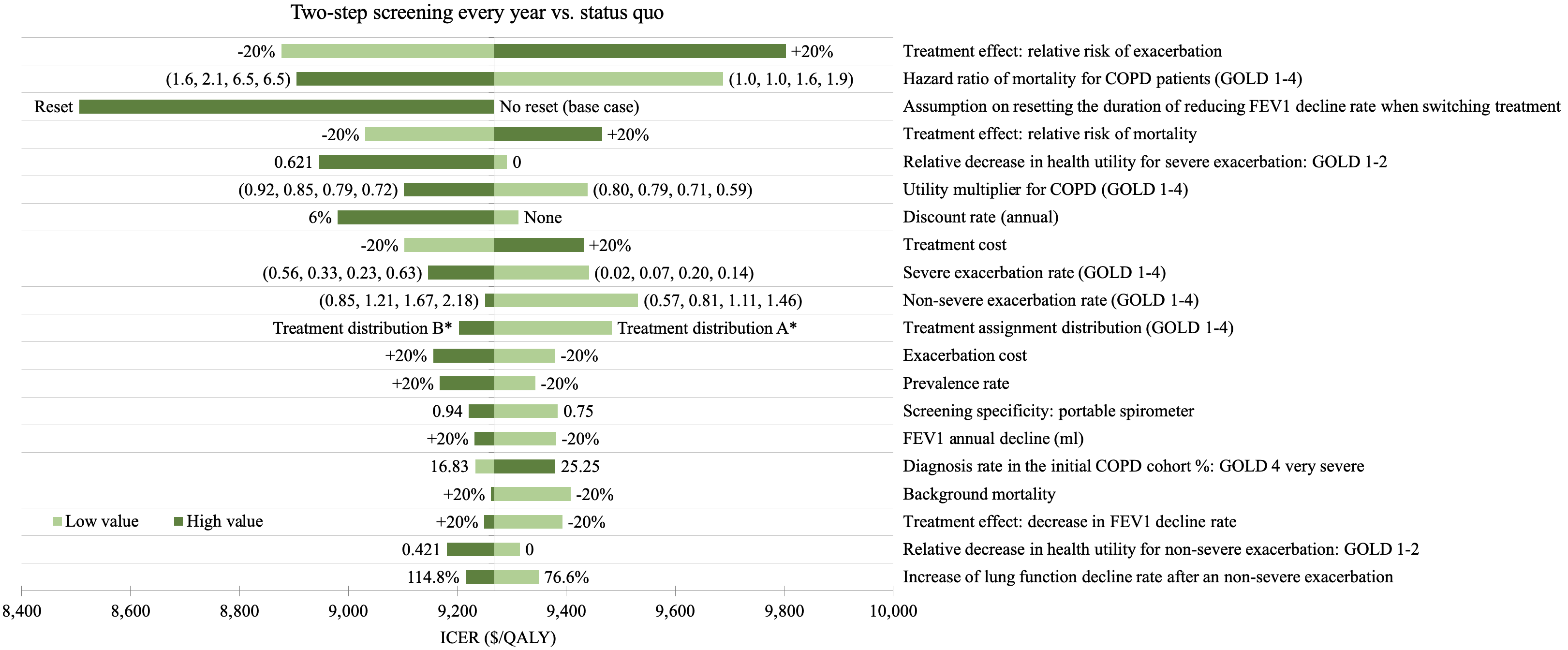


* Treatment distribution A: LABA, LABA, LAMA, and LABA/LAMA for GOLD stages 1-4, respectively; treatment distribution B: LAMA, LABA/LAMA, LABA/LAMA/ICS, and LABA/LAMA/ICS for GOLD stages 1-4, respectively.

Figure S9. Tornado diagram for one-way deterministic sensitivity analysis: two-step screening every year vs. one-step screening every year.

**
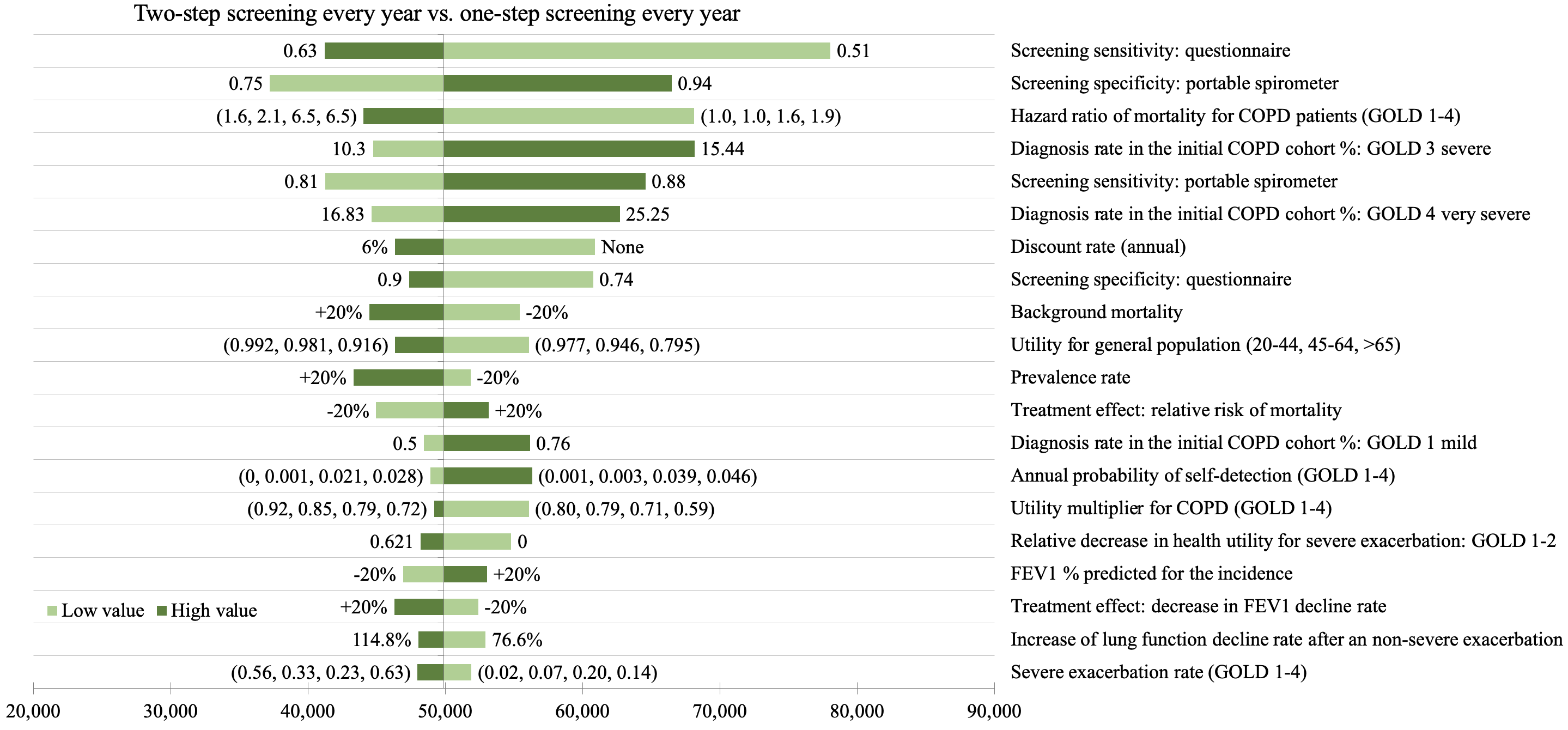
**

**Figure S10. Cost-effectiveness acceptability curves for all COPD screening policies.**


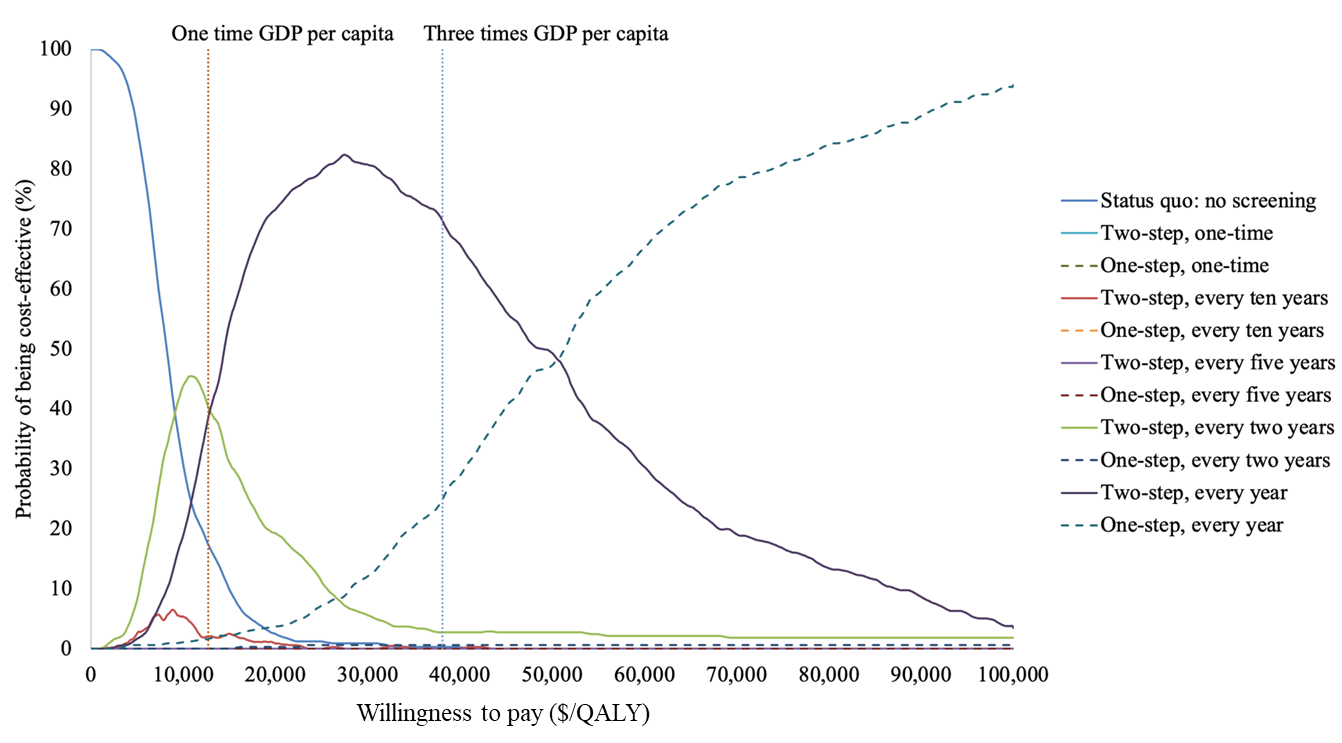


**Table S10. Cost for all screening policies in a cohort of one million individuals over a lifetime horizon.**

| **Screening policy** | | **Total cost (US$ millions)** | **Screening cost (US$ millions)** | **Diagnostic cost (US$ millions)** | **Exacerbation cost (US$ millions)** | **Pneumonia cost (US$ millions)** | **Treatment cost (US$ millions)** | **Maintenance cost (US$ millions)** |
| --- | --- | --- | --- | --- | --- | --- | --- | --- |
| **Frequency** | **Method** |  |  |  |  |  |  |  |
| Status quo | No screening | 1,373.7 | 0.0 | 0.0 | 872.2 | 7.4 | 36.7 | 457.4 |
| One-time | Two-step | 1,411.0 | 5.4 | 0.8 | 865.0 | 7.4 | 75.1 | 457.3 |
| One-time | One-step | 1,418.4 | 4.7 | 2.4 | 863.6 | 7.5 | 83.1 | 457.1 |
| Every ten years | Two-step | 1,430.2 | 6.7 | 1.4 | 863.1 | 7.4 | 93.8 | 457.7 |
| Every ten years | One-step | 1,438.7 | 5.6 | 4.3 | 861.2 | 7.4 | 103.0 | 457.1 |
| Every five years | Two-step | 1,450.3 | 8.6 | 2.2 | 858.9 | 7.5 | 115.3 | 457.8 |
| Every five years | One-step | 1,464.7 | 6.5 | 7.7 | 857.1 | 7.5 | 128.5 | 457.4 |
| Every two years | Two-step | 1,494.2 | 14.1 | 4.1 | 850.6 | 7.6 | 160.6 | 457.3 |
| Every two years | One-step | 1,511.8 | 9.5 | 15.6 | 849.8 | 7.6 | 172.4 | 457.0 |
| Every year | Two-step | 1,538.4 | 23.3 | 6.9 | 845.1 | 7.7 | 199.2 | 456.2 |
| Every year | One-step | 1,561.2 | 14.6 | 30.4 | 841.8 | 7.6 | 210.2 | 456.7 |

Table S11. Results of two-way sensitivity analysis for linkage to care in a cohort of one million individuals over a lifetime horizon.

| **Probability of Follow-up diagnosis** | **Probability of Treatment uptake** | **The most cost-effective policy** | **Status quo** | | **The most cost-effective policy** | | **ICER $/QALY (vs. status quo)** |
| --- | --- | --- | --- | --- | --- | --- | --- |
|  |  |  | **QALY (millions)** | **Cost (US$ millions)** | **Incremental QALY** | **Incremental cost (US$ millions)** |  |
| 30% | 20% | Two-step, every year | 16.191 | 1,360 | 8,100 | 100,905,127 | 12,458 |
| 30% | 30% | Two-step, every year | 16.192 | 1,374 | 10,630 | 102,298,817 | 9,624 |
| 30% | 40% | Two-step, every year | 16.193 | 1,384 | 12,079 | 106,761,442 | 8,838 |
| 30% | 50% | Two-step, every year | 16.194 | 1,396 | 14,716 | 124,784,872 | 8,480 |
| 30% | 60% | One-step, every year | 16.196 | 1,408 | 19,425 | 177,363,224 | 9,131 |
| 30% | 70% | One-step, every year | 16.196 | 1,417 | 20,643 | 185,178,376 | 8,971 |
| 30% | 80% | One-step, every year | 16.198 | 1,428 | 22,526 | 198,991,555 | 8,834 |
| 40% | 20% | Two-step, every year | 16.191 | 1,360 | 14,603 | 154,529,266 | 10,582 |
| 40%  (base case) | 30%  (base case) | Two-step, every year | 16.192 | 1,374 | 17,768 | 164,658,583 | 9,267 |
| 40% | 40% | Two-step, every year | 16.193 | 1,384 | 18,885 | 164,658,583 | 8,719 |
| 40% | 50% | Two-step, every year | 16.194 | 1,396 | 20,823 | 173,653,241 | 8,339 |
| 40% | 60% | One-step, every year | 16.196 | 1,408 | 25,146 | 227,221,365 | 9,036 |
| 40% | 70% | One-step, every year | 16.196 | 1,417 | 28,702 | 256,558,052 | 8,939 |
| 40% | 80% | One-step, every year | 16.198 | 1,428 | 31,055 | 273,463,551 | 8,806 |
| 50% | 20% | Two-step, every year | 16.191 | 1,360 | 21,561 | 211,961,548 | 9,831 |
| 50% | 30% | Two-step, every year | 16.192 | 1,374 | 23,543 | 215,671,792 | 9,161 |
| 50% | 40% | Two-step, every year | 16.193 | 1,384 | 25,645 | 223,382,036 | 8,711 |
| 50% | 50% | Two-step, every year | 16.194 | 1,396 | 27,985 | 233,057,174 | 8,328 |
| 50% | 60% | Two-step, every year | 16.196 | 1,408 | 30,451 | 247,104,811 | 8,115 |
| 50% | 70% | One-step, every year | 16.196 | 1,417 | 33,960 | 303,132,939 | 8,926 |
| 50% | 80% | One-step, every year | 16.198 | 1,428 | 36,677 | 322,258,605 | 8,786 |
| 60% | 20% | Two-step, every two years | 16.191 | 1,360 | 25,771 | 186,878,693 | 7,252 |
| 60% | 30% | Two-step, every year | 16.192 | 1,374 | 29,912 | 271,933,177 | 9,091 |
| 60% | 40% | Two-step, every year | 16.193 | 1,384 | 32,411 | 278,576,231 | 8,595 |
| 60% | 50% | Two-step, every year | 16.194 | 1,396 | 34,619 | 287,371,543 | 8,301 |
| 60% | 60% | Two-step, every year | 16.196 | 1,408 | 36,263 | 293,768,713 | 8,101 |
| 60% | 70% | Two-step, every year | 16.196 | 1,417 | 36,953 | 297,544,202 | 8,052 |
| 60% | 80% | One-step, every year | 16.198 | 1,428 | 40,239 | 352,059,904 | 8,749 |
| 70% | 20% | Two-step, every two years | 16.191 | 1,360 | 31,912 | 229,751,953 | 7,200 |
| 70% | 30% | Two-step, every year | 16.192 | 1,374 | 36,534 | 331,407,609 | 9,071 |
| 70% | 40% | Two-step, every year | 16.193 | 1,384 | 39,186 | 333,433,085 | 8,509 |
| 70% | 50% | Two-step, every year | 16.194 | 1,396 | 42,146 | 349,346,224 | 8,289 |
| 70% | 60% | Two-step, every year | 16.196 | 1,408 | 43,475 | 351,106,244 | 8,076 |
| 70% | 70% | Two-step, every year | 16.196 | 1,417 | 44,385 | 356,809,663 | 8,039 |
| 70% | 80% | One-step, every year | 16.198 | 1,428 | 47,551 | 415,657,054 | 8,741 |
| 80% | 20% | Two-step, every two years | 16.191 | 1,360 | 36,297 | 261,124,896 | 7,194 |
| 80% | 30% | Two-step, every year | 16.192 | 1,374 | 43,981 | 397,229,131 | 9,032 |
| 80% | 40% | Two-step, every year | 16.193 | 1,384 | 47,239 | 398,678,176 | 8,440 |
| 80% | 50% | Two-step, every year | 16.194 | 1,396 | 48,462 | 400,538,240 | 8,265 |
| 80% | 60% | Two-step, every year | 16.196 | 1,408 | 51,582 | 413,689,769 | 8,020 |
| 80% | 70% | Two-step, every year | 16.196 | 1,417 | 52,786 | 422,867,298 | 8,011 |
| 80% | 80% | Two-step, every year | 16.198 | 1,428 | 54,764 | 429,842,636 | 7,849 |
| 90% | 20% | Two-step, every two years | 16.191 | 1,360 | 41,417 | 296,783,411 | 7,166 |
| 90% | 30% | Two-step, every two years | 16.192 | 1,374 | 45,429 | 302,110,473 | 6,650 |
| 90% | 40% | Two-step, every year | 16.193 | 1,384 | 51,724 | 435,981,596 | 8,429 |
| 90% | 50% | Two-step, every year | 16.194 | 1,396 | 54,884 | 450,217,021 | 8,203 |
| 90% | 60% | Two-step, every year | 16.196 | 1,408 | 57,139 | 457,169,139 | 8,001 |
| 90% | 70% | Two-step, every year | 16.196 | 1,417 | 58,612 | 468,896,000 | 8,000 |
| 90% | 80% | Two-step, every year | 16.198 | 1,428 | 60,385 | 471,926,887 | 7,815 |
| 100% | 20% | Two-step, every two years | 16.191 | 1,360 | 46,598 | 333,121,489 | 7,149 |
| 100% | 30% | Two-step, every two years | 16.192 | 1,374 | 50,456 | 335,152,544 | 6,642 |
| 100% | 40% | Two-step, every year | 16.193 | 1,384 | 57,785 | 484,700,580 | 8,388 |
| 100% | 50% | Two-step, every year | 16.194 | 1,396 | 60,264 | 490,850,280 | 8,145 |
| 100% | 60% | Two-step, every year | 16.196 | 1,408 | 63,261 | 505,265,607 | 7,987 |
| 100% | 70% | Two-step, every year | 16.196 | 1,417 | 65,054 | 515,748,112 | 7,928 |
| 100% | 80% | Two-step, every year | 16.198 | 1,428 | 68,807 | 536,694,600 | 7,800 |

**Table S12. Detailed results for one-way sensitivity analysis.**

|  |  | **A: Status quo** | | **B: Two-step every year** | | **C: One-step every year** | | **Cost-effectiveness** | |
| --- | --- | --- | --- | --- | --- | --- | --- | --- | --- |
| **Parameter** | **Value** | **QALY (millions)** | **Cost (US$ millions)** | **QALY (millions)** | **Cost (US$ millions** | **QALY (millions)** | **Cost (US$ millions** | **ICER $/QALY (B vs. A)** | **ICER $/QALY (C vs. B)** |
| Prevalence rate | -20% | 16.203 | 1,348 | 16.221 | 1,512 | 16.221 | 1,526 | 9,343 | 51,853 |
| Prevalence rate | +20% | 16.179 | 1,399 | 16.197 | 1,566 | 16.198 | 1,579 | 9,168 | 43,316 |
| Incidence rate | -20% | 16.193 | 1,365 | 16.210 | 1,523 | 16.211 | 1,584 | 9,328 | 50,309 |
| Incidence rate | +20% | 16.188 | 1,378 | 16.208 | 1,562 | 16.209 | 1,635 | 9,251 | 49,254 |
| Background mortality | -20% | 16.233 | 1,383 | 16.251 | 1,549 | 16.251 | 1,564 | 9,408 | 55,423 |
| Background mortality | +20% | 16.148 | 1,366 | 16.165 | 1,530 | 16.166 | 1,546 | 9,262 | 44,463 |
| Diagnosis rate in the initial COPD cohort %: GOLD 1 mild | 0.5 | 16.192 | 1,374 | 16.210 | 1,538 | 16.210 | 1,566 | 9,315 | 48,459 |
| Diagnosis rate in the initial COPD cohort %: GOLD 1 mild | 0.76 | 16.192 | 1,374 | 16.210 | 1,539 | 16.210 | 1,567 | 9,301 | 56,162 |
| Diagnosis rate in the initial COPD cohort %: GOLD 2 moderate | 1.6 | 16.192 | 1,374 | 16.210 | 1,539 | 16.210 | 1,567 | 9,254 | 49,004 |
| Diagnosis rate in the initial COPD cohort %: GOLD 2 moderate | 2.4 | 16.192 | 1,374 | 16.210 | 1,539 | 16.210 | 1,568 | 9,299 | 52,138 |
| Diagnosis rate in the initial COPD cohort %: GOLD 3 severe | 10.3 | 16.192 | 1,374 | 16.210 | 1,539 | 16.210 | 1,565 | 9,262 | 44,747 |
| Diagnosis rate in the initial COPD cohort %: GOLD 3 severe | 15.44 | 16.193 | 1,374 | 16.210 | 1,539 | 16.211 | 1,569 | 9,393 | 68,161 |
| Diagnosis rate in the initial COPD cohort %: GOLD 4 very severe | 16.83 | 16.192 | 1,374 | 16.210 | 1,539 | 16.210 | 1,564 | 9,233 | 44,643 |
| Diagnosis rate in the initial COPD cohort %: GOLD 4 very severe | 25.25 | 16.192 | 1,375 | 16.210 | 1,542 | 16.210 | 1,568 | 9,380 | 62,722 |
| FEV1 % predicted for the initial population | -20% | 16.181 | 1,400 | 16.199 | 1,565 | 16.199 | 1,588 | 9,243 | 48,978 |
| FEV1 % predicted for the initial population | +20% | 16.201 | 1,353 | 16.219 | 1,517 | 16.219 | 1,543 | 9,297 | 50,826 |
| FEV1 % predicted for the incidence | -20% | 16.194 | 1,365 | 16.211 | 1,523 | 16.212 | 1,555 | 9,328 | 46,918 |
| FEV1 % predicted for the incidence | +20% | 16.188 | 1,378 | 16.208 | 1,562 | 16.208 | 1,566 | 9,260 | 53,052 |
| FEV1 annual decline (ml) | -20% | 16.195 | 1,366 | 16.213 | 1,533 | 16.214 | 1,556 | 9,381 | 50,546 |
| FEV1 annual decline (ml) | +20% | 16.189 | 1,381 | 16.207 | 1,545 | 16.207 | 1,569 | 9,232 | 48,842 |
| Non-severe exacerbation rate (GOLD 1-4) | (0.57, 0.81, 1.11, 1.46) | 16.193 | 1,371 | 16.211 | 1,540 | 16.211 | 1,565 | 9,532 | 51,555 |
| Non-severe exacerbation rate (GOLD 1-4) | (0.85, 1.21, 1.67, 2.18) | 16.191 | 1,377 | 16.209 | 1,542 | 16.209 | 1,569 | 9,251 | 48,104 |
| Severe exacerbation rate (GOLD 1-4) | (0.02, 0.07, 0.20, 0.14) | 16.192 | 1,357 | 16.210 | 1,525 | 16.210 | 1,551 | 9,442 | 51,909 |
| Severe exacerbation rate (GOLD 1-4) | (0.56, 0.33, 0.23, 0.63) | 16.191 | 1,387 | 16.209 | 1,552 | 16.210 | 1,577 | 9,146 | 47,986 |
| Increase of lung function decline rate after an non-severe exacerbation | 76.6% | 16.194 | 1,369 | 16.212 | 1,536 | 16.213 | 1,562 | 9,349 | 52,940 |
| Increase of lung function decline rate after an non-severe exacerbation | 114.8% | 16.190 | 1,379 | 16.208 | 1,542 | 16.208 | 1,568 | 9,215 | 48,034 |
| Increase of lung function decline rate after an severe exacerbation | 76.6% | 16.192 | 1,373 | 16.210 | 1,538 | 16.211 | 1,572 | 9,311 | 51,374 |
| Increase of lung function decline rate after an severe exacerbation | 114.8% | 16.192 | 1,375 | 16.209 | 1,539 | 16.210 | 1,572 | 9,278 | 47,694 |
| Annual probability of self-detection (GOLD 1-4) | (0, 0.001, 0.021, 0.028) | 16.192 | 1,374 | 16.210 | 1,538 | 16.210 | 1,563 | 9,284 | 48,908 |
| Annual probability of self-detection (GOLD 1-4) | (0.001, 0.003, 0.039, 0.046) | 16.192 | 1,375 | 16.210 | 1,539 | 16.210 | 1,565 | 9,325 | 56,321 |
| Hazard ratio of mortality for COPD patients (GOLD 1-4) | (1.0, 1.0, 1.6, 1.9) | 16.242 | 1,374 | 16.259 | 1,543 | 16.260 | 1,564 | 9,688 | 68,139 |
| Hazard ratio of mortality for COPD patients (GOLD 1-4) | (1.6, 2.1, 6.5, 6.5) | 16.540 | 1,373 | 16.559 | 1,538 | 16.559 | 1,559 | 8,905 | 44,042 |
| Screening sensitivity: questionnaire | 0.51 | 16.192 | 1,374 | 16.210 | 1,539 | 16.210 | 1,564 | 9,305 | 78,058 |
| Screening sensitivity: questionnaire | 0.63 | 16.192 | 1,374 | 16.210 | 1,539 | 16.210 | 1,565 | 9,204 | 41,217 |
| Screening specificity: questionnaire | 0.74 | 16.192 | 1,374 | 16.210 | 1,540 | 16.210 | 1,566 | 9,312 | 60,770 |
| Screening specificity: questionnaire | 0.9 | 16.192 | 1,374 | 16.210 | 1,539 | 16.210 | 1,565 | 9,263 | 47,352 |
| Screening sensitivity: portable spirometer | 0.81 | 16.192 | 1,374 | 16.210 | 1,539 | 16.210 | 1,562 | 9,316 | 41,281 |
| Screening sensitivity: portable spirometer | 0.88 | 16.192 | 1,374 | 16.210 | 1,539 | 16.210 | 1,562 | 9,233 | 64,587 |
| Screening specificity: portable spirometer | 0.75 | 16.192 | 1,374 | 16.210 | 1,540 | 16.210 | 1,562 | 9,384 | 37,225 |
| Screening specificity: portable spirometer | 0.94 | 16.192 | 1,374 | 16.210 | 1,539 | 16.210 | 1,562 | 9,221 | 66,493 |
| Treatment effect: decrease in FEV1 decline rate | -20% | 16.192 | 1,374 | 16.210 | 1,539 | 16.210 | 1,570 | 9,393 | 52,394 |
| Treatment effect: decrease in FEV1 decline rate | +20% | 16.192 | 1,374 | 16.210 | 1,538 | 16.210 | 1,570 | 9,249 | 46,290 |
| Treatment effect: relative risk of exacerbation | -20% | 16.192 | 1,373 | 16.210 | 1,535 | 16.211 | 1,572 | 8,877 | 49,172 |
| Treatment effect: relative risk of exacerbation | +20% | 16.192 | 1,374 | 16.209 | 1,544 | 16.210 | 1,581 | 9,803 | 51,251 |
| Treatment effect: relative risk of mortality | -20% | 16.192 | 1,375 | 16.211 | 1,541 | 16.211 | 1,568 | 9,031 | 44,966 |
| Treatment effect: relative risk of mortality | +20% | 16.192 | 1,374 | 16.209 | 1,537 | 16.210 | 1,561 | 9,465 | 53,182 |
| Utility for general population (20-44, 45-64, >65) | (0.977, 0.946, 0.795) | 16.192 | 1,374 | 16.209 | 1,538 | 16.210 | 1,562 | 9,315 | 56,083 |
| Utility for general population (20-44, 45-64, >65) | (0.992, 0.981, 0.916) | 16.423 | 1,374 | 16.441 | 1,538 | 16.442 | 1,562 | 9,262 | 46,351 |
| Utility multiplier for COPD (GOLD 1-4) | (0.80, 0.79, 0.71, 0.59) | 15.841 | 1,374 | 15.858 | 1,538 | 15.859 | 1,562 | 9,439 | 56,083 |
| Utility multiplier for COPD (GOLD 1-4) | (0.92, 0.85, 0.79, 0.72) | 16.543 | 1,374 | 16.561 | 1,538 | 16.561 | 1,562 | 9,102 | 49,197 |
| Relative decrease in health utility for non-severe exacerbation: GOLD 1-2 | 0 | 16.195 | 1,374 | 16.213 | 1,538 | 16.213 | 1,562 | 9,315 | 51,296 |
| Relative decrease in health utility for non-severe exacerbation: GOLD 1-2 | 0.421 | 16.185 | 1,374 | 16.203 | 1,538 | 16.204 | 1,562 | 9,181 | 49,197 |
| Relative decrease in health utility for severe exacerbation: GOLD 1-2 | 0 | 16.205 | 1,374 | 16.223 | 1,538 | 16.224 | 1,562 | 9,291 | 54,805 |
| Relative decrease in health utility for severe exacerbation: GOLD 1-2 | 0.621 | 16.180 | 1,374 | 16.199 | 1,538 | 16.199 | 1,562 | 8,947 | 48,210 |
| Screening cost: questionnaire | -20% | 16.192 | 1,374 | 16.210 | 1,538 | 16.210 | 1,561 | 9,254 | 49,890 |
| Screening cost: questionnaire | +20% | 16.192 | 1,374 | 16.210 | 1,539 | 16.210 | 1,562 | 9,305 | 50,676 |
| Screening cost: portable spirometer | -20% | 16.192 | 1,374 | 16.210 | 1,538 | 16.210 | 1,562 | 9,261 | 52,641 |
| Screening cost: portable spirometer | +20% | 16.192 | 1,374 | 16.210 | 1,540 | 16.210 | 1,562 | 9,351 | 49,190 |
| Screening cost: spirometry test | -20% | 16.192 | 1,374 | 16.210 | 1,538 | 16.210 | 1,561 | 9,260 | 48,760 |
| Screening cost: spirometry test | +20% | 16.192 | 1,374 | 16.210 | 1,539 | 16.210 | 1,562 | 9,315 | 50,070 |
| Maintenance cost | -20% | 16.192 | 1,365 | 16.210 | 1,530 | 16.210 | 1,553 | 9,269 | 49,862 |
| Maintenance cost | +20% | 16.192 | 1,382 | 16.210 | 1,548 | 16.210 | 1,571 | 9,323 | 50,231 |
| Treatment cost | -20% | 16.192 | 1,373 | 16.210 | 1,535 | 16.210 | 1,557 | 9,103 | 49,596 |
| Treatment cost | +20% | 16.192 | 1,374 | 16.210 | 1,542 | 16.210 | 1,565 | 9,432 | 50,181 |
| Exacerbation cost | -20% | 16.192 | 1,358 | 16.210 | 1,525 | 16.210 | 1,548 | 9,379 | 50,144 |
| Exacerbation cost | +20% | 16.192 | 1,390 | 16.210 | 1,552 | 16.210 | 1,575 | 9,156 | 49,318 |
| Discount rate (annual) | None | 24.387 | 1,925 | 24.417 | 2,209 | 24.418 | 2,223 | 9,312 | 60,916 |
| Discount rate (annual) | 6% | 11.656 | 1,047 | 11.670 | 1,165 | 11.670 | 1,167 | 8,980 | 46,338 |
| Treatment assignment distribution (GOLD 1-4) | Treatment distribution A* | 16.188 | 1,376 | 16.201 | 1,499 | 16.202 | 1,525 | 9,483 | 50,838 |
| Treatment assignment distribution (GOLD 1-4) | Treatment distribution B* | 16.192 | 1,384 | 16.216 | 1,605 | 16.216 | 1,625 | 9,203 | 47,444 |
| Assumption on resetting the duration of reducing FEV1 decline rate when switching treatment | No reset (base case) | 16.192 | 1,374 | 16.210 | 1,538 | 16.210 | 1,561 | 9,267 | 49,889 |
| Assumption on resetting the duration of reducing FEV1 decline rate when switching treatment | Reset | 16.196 | 1,373 | 16.215 | 1,538 | 16.216 | 1,561 | 8,506 | 47,630 |

* Treatment distribution A: LABA, LABA, LAMA, and LABA/LAMA for GOLD stages 1-4, respectively; treatment distribution B: LAMA, LABA/LAMA, LABA/LAMA/ICS, and LABA/LAMA/ICS for GOLD stages 1-4, respectively.

**Table S13.** **Results of additional scenario analyses in screening accuracy of different screening tests.**

|  |  |  | **A: Two-step every year** | | **B: One-step every year** | | **Cost-effectiveness** | |
| --- | --- | --- | --- | --- | --- | --- | --- | --- |
|  | **Sensitivity (%)** | **Specificity (%)** | **QALY** | **Cost (US$)** | **QALY** | **Cost (US$)** | **ICER $/QALY**  **(A vs. status quo)** | **ICER $/QALY (B vs. A)** |
| **Screening questionnaires** |  |  |  |  |  |  |  |  |
| COPD-SQ (base case)^56^ | 56.6 | 82.1 | 16,209,583.7 | 1,538,358,870.6 | 16,210,042.3 | 1,561,238,389.0 | 9,267 | 49,889 |
| COPD-SQ^57^ | 60.6 | 85.2 | 16,209,675.4 | 1,538,467,530.4 | 16,210,122.5 | 1,561,289,773.3 | 9,226 | 51,038 |
| COPD-SQ^58^ | 61.7 | 68.9 | 16,209,700.6 | 1,538,497,733.3 | 16,210,144.6 | 1,561,630,081.1 | 9,215 | 52,099 |
| COPD-SQ^59^ | 59.8 | 62.0 | 16,209,658.0 | 1,538,447,309.1 | 16,210,107.3 | 1,561,710,368.2 | 9,234 | 51,772 |
| COPD-PS^62^ | 74.5 | 64.4 | 16,209,993.8 | 1,538,845,715.6 | 16,210,401.2 | 1,562,069,296.5 | 9,085 | 57,010 |
| COPD-PS^63^ | 66.0 | 86.0 | 16,209,799.1 | 1,538,614,284.7 | 16,210,230.8 | 1,561,423,512.3 | 9,171 | 52,836 |
| COPD-PS^59^ | 61.4 | 65.0 | 16,209,694.2 | 1,538,490,196.9 | 16,210,139.0 | 1,561,697,281.9 | 9,217 | 52,173 |
| COPD-PS^58^ | 75.6 | 58.0 | 16,210,019.0 | 1,538,875,732.4 | 16,210,423.3 | 1,562,220,772.5 | 9,074 | 57,755 |
| COPD-PS^56^ | 40.3 | 89.9 | 16,209,210.3 | 1,537,915,698.8 | 16,209,715.6 | 1,560,640,447.2 | 9,441 | 44,974 |
| COPD-PS^61^ | 84.4 | 60.7 | 16,210,220.7 | 1,539,114,861.3 | 16,210,599.7 | 1,562,412,165.2 | 8,988 | 61,468 |
| COPD-CPHS^64^ | 69.0 | 72.0 | 16,209,867.8 | 1,538,696,085.5 | 16,210,290.9 | 1,561,772,491.7 | 9,140 | 54,540 |
| **Portable spirometers^60^** |  |  |  |  |  |  |  |  |
| Pooled estimate (base case) | 85 | 85 | 16,209,583.7 | 1,538,358,870.6 | 16,210,042.3 | 1,561,238,389.0 | 9,267 | 49,889 |
| COPD-6 | 84 | 85 | 16,209,541.6 | 1,538,268,137.1 | 16,210,042.3 | 1,561,238,389.0 | 9,284 | 45,872 |
| Piko-6 | 89 | 88 | 16,209,635.4 | 1,538,761,117.7 | 16,210,042.3 | 1,561,238,389.0 | 9,263 | 55,229 |
| PEF | 77 | 83 | 16,209,489.8 | 1,538,004,510.0 | 16,210,042.3 | 1,561,238,389.0 | 9,297 | 42,050 |
| Hi-checker | 75 | 86 | 16,209,480.4 | 1,538,002,098.3 | 16,210,042.3 | 1,561,238,389.0 | 9,301 | 41,348 |
| IQ-spiro | 94 | 92 | 16,209,706.5 | 1,539,101,321.1 | 16,210,042.3 | 1,561,238,389.0 | 9,245 | 65,915 |
| SP10BT | 83 | 75 | 16,209,538.9 | 1,538,327,532.6 | 16,210,042.3 | 1,561,238,389.0 | 9,289 | 45,509 |
| Medikro SpiroStar | 92 | 84 | 16,209,678.6 | 1,539,037,480.5 | 16,210,042.3 | 1,561,238,389.0 | 9,256 | 61,029 |
| MS01 Micro spirometer | 89 | 50 | 16,209,635.7 | 1,539,067,791.8 | 16,210,042.3 | 1,561,238,389.0 | 9,280 | 54,522 |
| Spirobank Smart | 83 | 92 | 16,209,538.7 | 1,538,314,491.8 | 16,210,042.3 | 1,561,238,389.0 | 9,288 | 45,519 |

Note: For status quo (without population-based COPD screening), our model estimated a total of 16,191,816.1 QALYs and a total cost of $1,373,700,287.8, which were the same across all rows and thus omitted in this table. We used the willingness-to-pay threshold of three times GDP per capita in China ($38,441) to determine the cost-effectiveness of the screening policy.

**Table S14.** **Results of additional scenario analysis for different treatment strategies.**

|  | **A: Status quo** | | **B: Two-step every year** | | **C: One-step every year** | | **Cost-effectiveness** | |
| --- | --- | --- | --- | --- | --- | --- | --- | --- |
| **Treatment assignment** | **QALY (millions)** | **Cost (US$ millions)** | **QALY (millions)** | **Cost (US$ millions)** | **QALY (millions)** | **Cost (US$ millions)** | **ICER $/QALY (B vs. A)** | **ICER $/QALY (C vs. B)** |
| Base case | 16.192 | 1,373.700 | 16.210 | 1,538.359 | 16.210 | 1,561.238 | 9,267 | 49,889 |
| LABA for all patients | 16.188 | 1,376.017 | 16.201 | 1,498.273 | 16.202 | 1,522.159 | 9,533 | 51,082 |
| LABA/LAMA/ICS for all patients | 16.192 | 1,384.158 | 16.225 | 1,683.958 | 16.226 | 1,710.310 | 8,988 | 42,704 |

Note: We considered the cost-effectiveness of the screening policy based on the willingness-to-pay threshold of three times GDP per capita in China ($38,441).

**Table S15.** **Results for additional scenario analysis with different LABA/LAMA and LABA/LAMA/ICS cost.**

|  |  |  | **A: Status quo** | | **B: Two-step every year** | | **C: One-step every year** | | **Cost-effectiveness** | |
| --- | --- | --- | --- | --- | --- | --- | --- | --- | --- | --- |
| **Treatment cost (US$ per month)** | **LABA**  **/LAMA** | **LABA**  **/LAMA**  **/ICS** | **QALY (millions)** | **Cost (US$ millions)** | **QALY (millions)** | **Cost (US$ millions)** | **QALY (millions)** | **Cost (US$ millions)** | **ICER $/QALY (B vs. A)** | **ICER $/QALY (C vs. B)** |
| Base case | 104.58 | 59.68 | 16.192 | 1,373.700 | 16.210 | 1,538.359 | 16.210 | 1,561.238 | 9,267 | 49,889 |
| Less expensive LABA/LAMA | 59.68 | 59.68 | 16.192 | 1,367.063 | 16.210 | 1,502.354 | 16.210 | 1,523.257 | 7,614 | 39,544 |
| More expensive LABA/LAMA/ICS | 104.58 | 104.58 | 16.192 | 1,378.016 | 16.210 | 1,561.769 | 16.210 | 1,585.934 | 10,342 | 45,712 |
|  | 104.58 | 156.87 | 16.192 | 1,383.042 | 16.210 | 1,589.033 | 16.210 | 1,614.693 | 11,594 | 48,543 |
|  | 104.58 | 209.16 | 16.192 | 1,388.068 | 16.210 | 1,616.297 | 16.210 | 1,643.453 | 12,845 | 51,373 |

Note: We considered the cost-effectiveness of the screening policy based on the willingness-to-pay threshold of three times GDP per capita in China ($38,441).

**REFERENCES**

1. Wang C, Xu J, Yang L, et al. Prevalence and risk factors of chronic obstructive pulmonary disease in China (the China Pulmonary Health [CPH] study): a national cross-sectional study. *The Lancet* 2018; **391**(10131): 1706-17.

2. National Bureau of Statistics of China. China Statistical Yearbook 2021. <http://www.stats.gov.cn/tjsj/ndsj/2021/indexch.htm> (accessed Jun 18 2023).

3. Institute for Health Metrics and Evaluation. Global Burden of Disease (GBD) study. <https://vizhub.healthdata.org/gbd-results/> (accessed Jun 18 2023).

4. Zhang J, Hu X, Shan G. Spirometry reference values for population aged 7–80 years in China. *Respirology* 2017; **22**(8): 1630-6.

5. Global Initiative for Chronic Obstructive Lung Disease. GLOBAL STRATEGY FOR PREVENTION, DIAGNOSIS AND MANAGEMENT OF COPD: 2024 Report. <https://goldcopd.org/2024-gold-report/> (accessed Feb 12 2024).

6. Solem CT, Sun SX, Sudharshan L, Macahilig C, Katyal M, Gao X. Exacerbation-related impairment of quality of life and work productivity in severe and very severe chronic obstructive pulmonary disease. *International journal of chronic obstructive pulmonary disease* 2013; **8**: 641.

7. Iheanacho I, Zhang S, King D, Rizzo M, Ismaila AS. Economic Burden of Chronic Obstructive Pulmonary Disease (COPD): A Systematic Literature Review. *Int J Chron Obstruct Pulmon Dis* 2020; **15**: 439-60.

8. Halpin DMG, Decramer M, Celli BR, Mueller A, Metzdorf N, Tashkin DP. Effect of a single exacerbation on decline in lung function in COPD. *Respir Med* 2017; **128**: 85-91.

9. WHO. Global Health Observatory data repository. <https://apps.who.int/gho/data/view.main.60340> (accessed Jun 18 2023).

10. Leivseth L, Nilsen TI, Mai XM, Johnsen R, Langhammer A. Lung function and respiratory symptoms in association with mortality: The HUNT Study. *COPD* 2014; **11**(1): 59-80.

11. Lan F, Li J, Yu C, et al. [Associations between airflow obstruction and total and cause-specific mortality in adults in China]. *Zhonghua Liu Xing Bing Xue Za Zhi* 2017; **38**(1): 13-9.

12. Mannino DM, Buist AS, Petty TL, Enright PL, Redd SC. Lung function and mortality in the United States: data from the First National Health and Nutrition Examination Survey follow up study. *Thorax* 2003; **58**(5): 388-93.

13. Mannino DM, Doherty DE, Sonia Buist A. Global Initiative on Obstructive Lung Disease (GOLD) classification of lung disease and mortality: findings from the Atherosclerosis Risk in Communities (ARIC) study. *Respir Med* 2006; **100**(1): 115-22.

14. Kong CY, McMahon PM, Gazelle GS. Calibration of disease simulation model using an engineering approach. *Value in Health* 2009; **12**(4): 521-9.

15. Stout NK, Knudsen AB, Kong CYJ, McMahon PM, Gazelle GS. Calibration methods used in cancer simulation models and suggested reporting guidelines. *Pharmacoeconomics* 2009; **27**(7): 533.

16. The China Pulmonary Health [CPH] study. The China Pulmonary Health [CPH] study dataset. 2018.

17. Rabe KF, Martinez FJ, Ferguson GT, et al. Triple inhaled therapy at two glucocorticoid doses in moderate-to-very-severe COPD. *New England Journal of Medicine* 2020; **383**(1): 35-48.

18. Lipson DA, Barnhart F, Brealey N, et al. Once-daily single-inhaler triple versus dual therapy in patients with COPD. *New England Journal of Medicine* 2018; **378**(18): 1671-80.

19. National Institute for Health and Care Excellence. Chronic obstructive pulmonary disease in over 16s: diagnosis and management. <https://www.nice.org.uk/guidance/ng115> (accessed Jun 18 2023).

20. Ram FS, Sestini P. Regular inhaled short acting beta2 agonists for the management of stable chronic obstructive pulmonary disease: Cochrane systematic review and meta-analysis. *Thorax* 2003; **58**(7): 580-4.

21. Appleton S, Jones T, Poole P, et al. Ipratropium bromide versus short acting beta-2 agonists for stable chronic obstructive pulmonary disease. *Cochrane Database Syst Rev* 2006; **2006**(2): CD001387.

22. Barrecheguren M, Monteagudo M, Ferrer J, et al. Treatment patterns in COPD patients newly diagnosed in primary care. A population-based study. *Respir Med* 2016; **111**: 47-53.

23. Price D, West D, Brusselle G, et al. Management of COPD in the UK primary-care setting: an analysis of real-life prescribing patterns. *Int J Chron Obstruct Pulmon Dis* 2014; **9**: 889-904.

24. Bourbeau J, Sebaldt RJ, Day A, et al. Practice patterns in the management of chronic obstructive pulmonary disease in primary practice: the CAGE study. *Can Respir J* 2008; **15**(1): 13-9.

25. Hoogendoorn M, Rutten-van Mölken MP, Hoogenveen RT, Al MJ, Feenstra TL. Developing and applying a stochastic dynamic population model for chronic obstructive pulmonary disease. *Value in Health* 2011; **14**(8): 1039-47.

26. Asukai Y, Baldwin M, Fonseca T, Gray A, Mungapen L, Price D. Improving clinical reality in chronic obstructive pulmonary disease economic modelling : development and validation of a micro-simulation approach. *Pharmacoeconomics* 2013; **31**(2): 151-61.

27. Qu S, You X, Liu T, et al. Cost-Effectiveness analysis of COPD screening programs in primary care for high-risk patients in China. *NPJ primary care respiratory medicine* 2021; **31**(1): 1-9.

28. Chronic Obstructive Pulmonary Disease Group of Chinese Thoracic Society, Chronic Obstructive Pulmonary Disease Committee of Chinese Association of Chest Physician. Guidelines for the diagnosis and management of chronic obstructive pulmonary disease (revised version 2021) (in Chinese). *Chin J Tuberc Respir Dis* 2021; **44**(03): 170-205.

29. Kew KM, Mavergames C, Walters JA. Long-acting beta2-agonists for chronic obstructive pulmonary disease. *Cochrane Database Syst Rev* 2013; (10): CD010177.

30. Oba Y, Keeney E, Ghatehorde N, Dias S. Dual combination therapy versus long‐acting bronchodilators alone for chronic obstructive pulmonary disease (COPD): a systematic review and network meta‐analysis. *Cochrane Database of Systematic Reviews* 2018; (12).

31. Mammen MJ, Pai V, Aaron SD, Nici L, Alhazzani W, Alexander PE. Dual LABA/LAMA Therapy versus LABA or LAMA Monotherapy for Chronic Obstructive Pulmonary Disease. A Systematic Review and Meta-analysis in Support of the American Thoracic Society Clinical Practice Guideline. *Ann Am Thorac Soc* 2020; **17**(9): 1133-43.

32. Cazzola M, Rogliani P, Calzetta L, Matera MG. Triple therapy versus single and dual long-acting bronchodilator therapy in COPD: a systematic review and meta-analysis. *Eur Respir J* 2018; **52**(6).

33. Koarai A, Sugiura H, Yamada M, et al. Treatment with LABA versus LAMA for stable COPD: a systematic review and meta-analysis. *BMC Pulm Med* 2020; **20**(1): 111.

34. Calzetta L, Ritondo BL, de Marco P, Cazzola M, Rogliani P. Evaluating triple ICS/LABA/LAMA therapies for COPD patients: a network meta-analysis of ETHOS, KRONOS, IMPACT, and TRILOGY studies. *Expert Rev Respir Med* 2021; **15**(1): 143-52.

35. Wu C, Gong Y, Wu J, et al. Chinese Version of the EQ-5D Preference Weights: Applicability in a Chinese General Population. *PLoS One* 2016; **11**(10): e0164334.

36. Moayeri F, Hsueh Y-SA, Clarke P, Dunt D. Do model-based studies in chronic obstructive pulmonary disease measure correct values of utility? A meta-analysis. *Value in Health* 2016; **19**(4): 363-73.

37. Brazier J, Ara R, Azzabi I, et al. Identification, Review, and Use of Health State Utilities in Cost-Effectiveness Models: An ISPOR Good Practices for Outcomes Research Task Force Report. *Value Health* 2019; **22**(3): 267-75.

38. Fan C, Dong C, Lin M, Chang J. The Cost Effectiveness Analysis Of Indacaterol Versus Tiotropium In A Chinese Medical Cost Setting. *Value in Health* 2014; **17**(3): A175.

39. Di M, Cao Y, Huang H. Investigation on the expenses of 248 adult patients with community-acquired pneumonia in Dongcheng District, Beijing. *Mod Prev Med (in Chinese)* 2014; **41**(14): 2560-2.

40. Mo X, Gai Tobe R, Wang L, et al. Cost-effectiveness analysis of different types of human papillomavirus vaccination combined with a cervical cancer screening program in mainland China. *BMC Infect Dis* 2017; **17**(1): 502.

41. Jiang H, Zhang P, Gu K, et al. Cost-effectiveness analysis of a community-based colorectal cancer screening program in Shanghai, China. *Front Public Health* 2022; **10**: 986728.

42. Huang QC, Ye D, Jiang XY, et al. [Cost-effectiveness analysis on colorectal cancer screening program]. *Zhonghua Liu Xing Bing Xue Za Zhi* 2017; **38**(1): 65-8.

43. Mo M, Zheng Y, Liu G, et al. [Cost-effectiveness analysis of two breast cancer screening modalities in Shanghai, China]. *Zhonghua Zhong Liu Za Zhi* 2015; **37**(12): 944-51.

44. Yuan J, Sun Y, Wang K, et al. Cost Effectiveness of Lung Cancer Screening With Low-Dose CT in Heavy Smokers in China. *Cancer Prev Res (Phila)* 2022; **15**(1): 37-44.

45. Beijing Municipal Medical Insurance Bureau. Notice on Regulating and Adjusting the Price of Imaging Medical Services. <http://ybj.beijing.gov.cn/zwgk/2020_zcwj/202106/t20210604_2407476.html> (accessed Jun 18 2023).

46. Beijing Municipal Medical Insurance Bureau. Notice on Regulating and Adjusting the Price of Clinical Diagnosis and Medical Services. <http://ybj.beijing.gov.cn/zwgk/2020_zcwj/202112/t20211231_2581064.html> (accessed Jun 18 2023).

47. Price D, Gray A, Gale R, et al. Cost-utility analysis of indacaterol in Germany: a once-daily maintenance bronchodilator for patients with COPD. *Respiratory medicine* 2011; **105**(11): 1635-47.

48. Borg S, Ericsson Å, Wedzicha J, et al. A computer simulation model of the natural history and economic impact of chronic obstructive pulmonary disease. *Value in Health* 2004; **7**(2): 153-67.

49. Hansen RN, Xu X, Sullivan SD. PRS18 A Dynamic Cohort Model of Chronic Obstructive Pulmonary Disease and Its Treatments. *Value in Health* 2012; **15**(4): A54.

50. Rutten-van Molken MP, Oostenbrink JB, Miravitlles M, Monz BU. Modelling the 5-year cost effectiveness of tiotropium, salmeterol and ipratropium for the treatment of chronic obstructive pulmonary disease in Spain. *Eur J Health Econ* 2007; **8**(2): 123-35.

51. Menn P, Leidl R, Holle R. A lifetime Markov model for the economic evaluation of chronic obstructive pulmonary disease. *Pharmacoeconomics* 2012; **30**(9): 825-40.

52. Samyshkin Y, Kotchie RW, Mörk A-C, Briggs AH, Bateman ED. Cost-effectiveness of roflumilast as an add-on treatment to long-acting bronchodilators in the treatment of COPD associated with chronic bronchitis in the United Kingdom. *The European Journal of Health Economics* 2014; **15**: 69-82.

53. Hoogendoorn M, Feenstra TL, Asukai Y, et al. Cost-effectiveness models for chronic obstructive pulmonary disease: cross-model comparison of hypothetical treatment scenarios. *Value Health* 2014; **17**(5): 525-36.

54. Zafari Z, Sin DD, Postma DS, et al. Individualized prediction of lung-function decline in chronic obstructive pulmonary disease. *CMAJ* 2016; **188**(14): 1004-11.

55. Zafari Z, Adibi A, Sadatsafavi M. Lung Function Predictor for COPD Patients. <https://resp.core.ubc.ca/ipress/FEV1Pred> (accessed Feb 12 2024).

56. Liu M, Yin D, Wang Y, et al. Comparing the Performance of Two Screening Questionnaires for Chronic Obstructive Pulmonary Disease in the Chinese General Population. *Int J Chron Obstruct Pulmon Dis* 2023; **18**: 541-52.

57. Zhou YM, Chen SY, Tian J, et al. Development and validation of a chronic obstructive pulmonary disease screening questionnaire in China. *Int J Tuberc Lung Dis* 2013; **17**(12): 1645-51.

58. Zhou J, Yu N, Li X, Wang W. Accuracy of Six Chronic Obstructive Pulmonary Disease Screening Questionnaires in the Chinese Population. *Int J Chron Obstruct Pulmon Dis* 2022; **17**: 317-27.

59. Yang S, Yin X, Zhang Y, et al. Efficacy of a Self-Designed Questionnaire for Community Screening of COPD. *Int J Chron Obstruct Pulmon Dis* 2022; **17**: 1381-91.

60. Zhou J, Li X, Wang X, Yu N, Wang W. Accuracy of portable spirometers in the diagnosis of chronic obstructive pulmonary disease A meta-analysis. *NPJ Prim Care Respir Med* 2022; **32**(1): 15.

61. Martinez FJ, Raczek AE, Seifer FD, et al. Development and initial validation of a self-scored COPD Population Screener Questionnaire (COPD-PS). *COPD* 2008; **5**(2): 85-95.

62. Zhan Z, Ma Y, Chen Y, et al. Validation of COPD Population Screener Questionnaire in Chinese Population: A National Multicenter Study. *Respiration* 2023; **102**(12): 995-1002.

63. Gu Y, Zhang Y, Wen Q, et al. Performance of COPD population screener questionnaire in COPD screening: a validation study and meta-analysis. *Ann Med* 2021; **53**(1): 1198-206.

64. Wang D, Fan G, Wu S, et al. Development and Validation of a Screening Questionnaire of COPD from a Large Epidemiological Study in China. *COPD* 2022; **19**(1): 118-24.

65. Briggs AH, Weinstein MC, Fenwick EA, et al. Model parameter estimation and uncertainty analysis: a report of the ISPOR-SMDM Modeling Good Research Practices Task Force Working Group-6. *Med Decis Making* 2012; **32**(5): 722-32.

66. Briggs A, Sculpher M, Claxton K. Decision modelling for health economic evaluation: Oup Oxford; 2006.

67. Jenkins CR, Jones PW, Calverley PM, et al. Efficacy of salmeterol/fluticasone propionate by GOLD stage of chronic obstructive pulmonary disease: analysis from the randomised, placebo-controlled TORCH study. *Respir Res* 2009; **10**: 59.

68. Bridevaux PO, Gerbase MW, Probst-Hensch NM, Schindler C, Gaspoz JM, Rochat T. Long-term decline in lung function, utilisation of care and quality of life in modified GOLD stage 1 COPD. *Thorax* 2008; **63**(9): 768-74.

69. Hoogendoorn M, Feenstra TL, Hoogenveen RT, Al M, Rutten-van Mölken M. Association between lung function and exacerbation frequency in patients with COPD. *International journal of chronic obstructive pulmonary disease* 2010; **5**: 435.

70. Mullerova H, Chigbo C, Hagan GW, et al. The natural history of community-acquired pneumonia in COPD patients: a population database analysis. *Respir Med* 2012; **106**(8): 1124-33.

71. Singh S, Amin AV, Loke YK. Long-term use of inhaled corticosteroids and the risk of pneumonia in chronic obstructive pulmonary disease: a meta-analysis. *Arch Intern Med* 2009; **169**(3): 219-29.

72. Tashkin DP, Celli B, Senn S, et al. A 4-year trial of tiotropium in chronic obstructive pulmonary disease. *New England Journal of Medicine* 2008; **359**(15): 1543-54.
